# Accurate Vertical Excitation Energies of BODIPY/Aza-BODIPY Derivatives from Excited-State Mean-Field Calculations

**DOI:** 10.1021/acs.jpca.2c04473

**Published:** 2022-09-29

**Authors:** Daniele Toffoli, Matteo Quarin, Giovanna Fronzoni, Mauro Stener

**Affiliations:** † Dipartimento di Scienze Chimiche e Farmaceutiche, Università degli Studi di Trieste, via L. Giorgieri 1, I-34127 Trieste, Italy; ‡ CNR-IOM, Istituto Officina dei Materiali, I-34149, Trieste, Italy

## Abstract

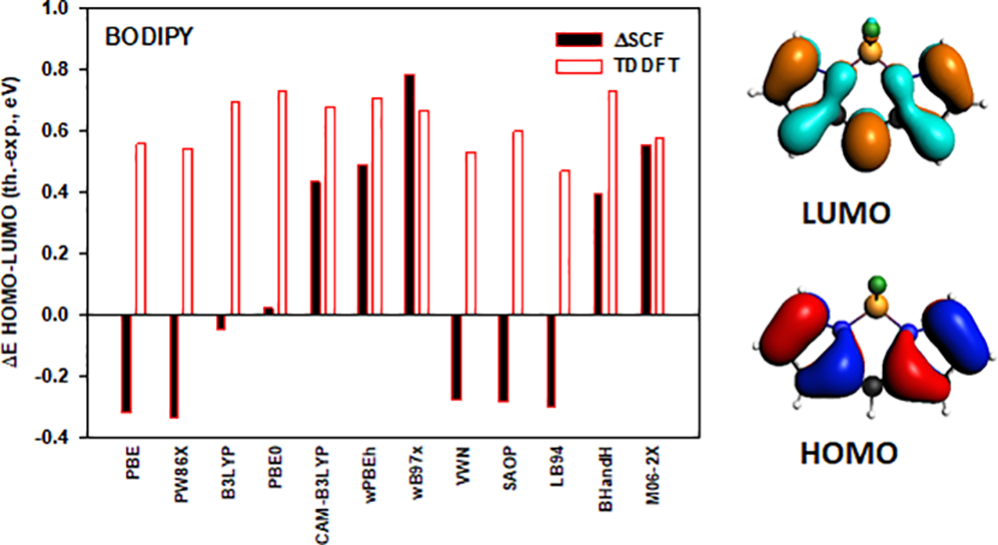

We report a benchmark study of vertical excitation energies and oscillator strengths for the HOMO → LUMO transitions of 17 boron–dipyrromethene (BODIPY) structures, showing a large variety of ring sizes and substituents. Results obtained at the time-dependent density functional theory (TDDFT) and at the delta-self-consistent-field (ΔSCF) by using 13 different exchange correlation kernels (within LDA, GGA, hybrid, and range-separated approximations) are benchmarked against the experimental excitation energies when available. It is found that the time-independent ΔSCF DFT method, when used in combination with hybrid PBE0 and B3LYP functionals, largely outperforms TDDFT and can be quite competitive, in terms of accuracy, with computationally more costly wave function based methods such as CC2 and CASPT2.

## I Introduction

**Figure 1:**
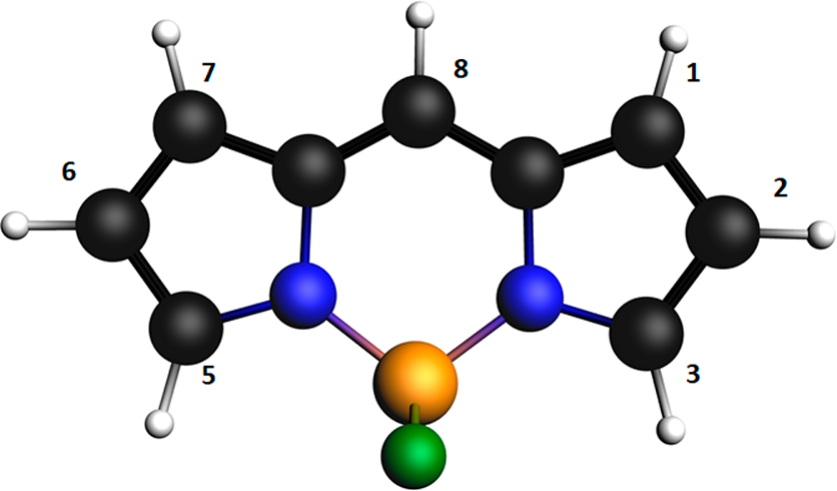
Structure of 8-H-BODIPY (4,4-difluoro-4-bora-3a,4a-diaza-*s*-indacene) together with a numbering scheme. The meso position is position 8. C atoms are in black color, N atoms in blue, H atoms in white, B atoms in orange, and F atoms in green.

Boron–dipyrromethenes (BODIPYs), together with their derivatives where the *meso*-carbon atom is substituted by a nitrogen (aza-BODIPYs), constitute an important class of organic dyes,^1^ due to the large number of potential applications in numerous fields (see Figure 1 for a sketch of the parent molecule 8-H-BODIPY). Research in these compounds (both experimental and *in-silico*) has sparkled in the past decade due to their interesting photophysical properties, such as intense and narrow fluorescence peaks with high quantum yield, and to the ease with which one can play with various substituents to influence their spectroscopic and photophysical signatures.^1,2^ BODIPYs find application in optoelectronics,^3^ electrochemistry and electroluminescence,^4^ nanomedicine,^5^ photodynamic therapy,^6−9^ photochemical signaling,^10^ dye-sensitized solar cells,^11−13^ and fluorescence and cellular imaging^14,15^

Since BODIPYs are medium to large molecules, time-dependent density functional theory^16,17^ (TDDFT) is the method of choice to investigate their spectroscopic properties, together with a selection of explicit wave function methods such as second-order approximate coupled-cluster,^18−20^ CC2, and multiconfigurational CAS-PT2.^21^ Compared to the latter methods, TDDFT still is computationally much more affordable, also because basis-set requirements for DFT are much less demanding than explicit wave function methods.^22^ Clearly the selection of a large enough active space proves crucial for the accuracy of CAS-PT2 estimates,^23^ and it can severely limit its applicability to even medium-size systems.

**Figure 2:**
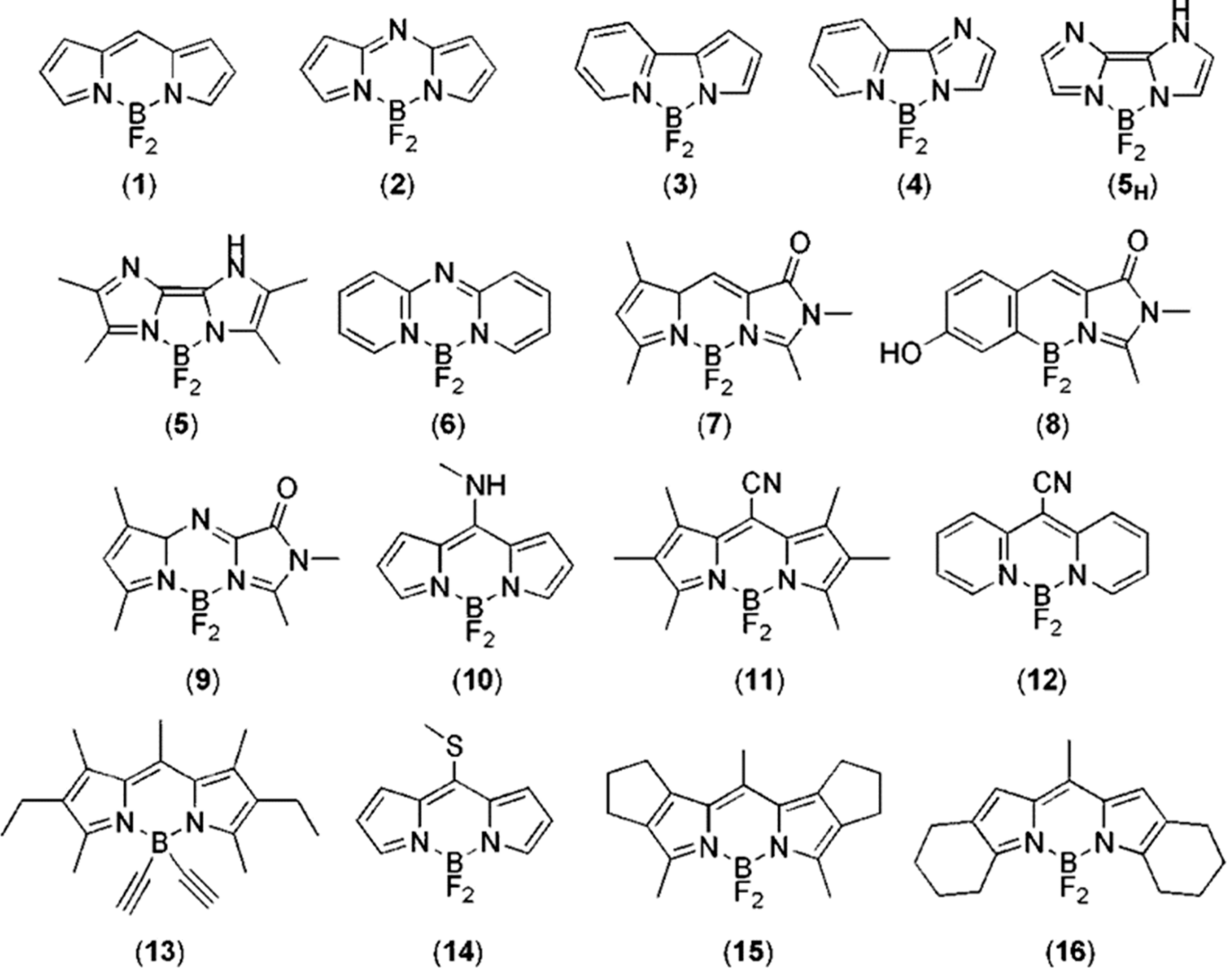
Chemical structures of BODIPYs and aza-BODIPYs considered in this work. Reprinted with permission from ref (39). Copyright 2015 American Chemical Society. The numbering follows that adopted by Momeni and Brown.^39^

Unfortunately in this class of molecules TDDFT excitation energies suffer from low accuracy (>0.3 eV), together with a strong dependence of the results on the particular xc potential used,^24^ when applied to even the lowest π → π* excitations.^25^ This observation applies both to excitation energies calculated within the vertical approximation, which is of widespread use in the dye community, and to computed adiabatic excitation energies with inclusion of solvent effects.^26,27^ TDDFT has well-known shortcomings when dealing with charge-transfer (CT) excitations^28,29^ and, due to the commonly adopted adiabatic approximation to the xc kernel,^17^ with excitations involving double-excitation character. While the former issue has been somewhat mitigated with the introduction of range-separated functionals, the latter cannot easily be cured within standard TDDFT. To bypass this problem, Boulanger et al.^30^ proposed a protocol where vertical excitation energies were calculated with the Bethe–Salpeter formalism or with the scaled opposite spin (SOS) CIS(D) method,^31^ which adds a perturbative correction for double excitations on top of a CIS calculation.^26^ Actually the importance of double excitations for the description of low-lying excited states is not restricted to BODIPYs and related families,^32,33^ but it appears to be related to the presence of boron in the molecular skeleton and has been recently evidenced in a series of works focused on near-edge X-ray absorption of boroxine-containing compounds.^34−37^ In particular, it was shown in refs (35 and 36) that while transition-state (TS) and TDDFT methods with a selection of xc potentials ranging from GGA to global, meta-separated, and range-separated hybrids fail to account for the correct intensity distribution of the lowest two spectral features, assigned to π* valence core excited states, a qualitatively correct description was obtained with a computationally inexpensive ΔSCF procedure.^35^ This observation, together with a recent publication by Worster et al.,^38^ which showed that ΔSCF is able to predict excitation energies with an accuracy competitive with and sometimes better than that of TDDFT, prompted us to benchmark ΔSCF against TDDFT on a series of 17 BODIPYs and aza-BODIPYs considered by Momeni and Brown^39^ and Feldt and Brown^40^ (see Figure 2) in the quest for an accurate yet efficient mean-field method that could be used for a fast screening of candidate dyes for a specific application. In this work, the accuracy of the ΔSCF method is explored and contrasted with that of TDDFT for a quite extensive range of xc potentials. It will be shown that the simple ΔSCF method, coupled with a rational choice of the xc potential, is able to predict vertical excitation energies of BODIPYs and aza-BODIPYs with an accuracy superior to TDDFT and comparable to that of the computationally much more demanding correlated wave function methods.

The plan of the paper is the following: in Section II we provide a brief overview of the theory and of the computational details. Section III is devoted to a discussion of the benchmark of vertical excitation energies against both TDDFT and experimental values, while our conclusions are presented in the final section, section IV.

## II Theory and Computational Details

Excitation energies and oscillator strengths have been calculated at the TDDFT level, in the nonrelativistic approximation, as implemented in the ADF code^41−43^ and within the adiabatic local density approximation^17^ (ALDA) to the exchange-correlation (xc) kernel.

In linear response TDDFT, excitation energies and intensities are obtained through the solution of the following eigenvalue equation by means of Davidson’s iterative algorithm:^44^
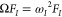
1Here the elements of the Ω matrix are given by
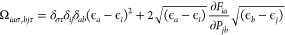
2

In eq 2, indices *i* and *j* run over the set of occupied molecular orbitals (MOs) in the KS ground-state, while indices *a* and *b* run over the set of virtual MOs; ε_*i*_ and ε_*a*_ are the KS molecular orbital energies. *F* and *P* represent the Fock matrix and the density matrix, respectively, whereas  are the elements of the so-called “coupling matrix”, *K*, which can be written as a sum of a Hartree (Coulomb) part plus the xc part as follows:
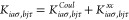
3

Eigenvalues ω_*I*_^2^ in eq 1 correspond to squared excitation energies, while the oscillator strengths are extracted from the eigenvectors *F*_*I*_ according to standard TDDFT.^17^

Excitation energies and oscillator strengths for the HOMO→ LUMO transition have been also calculated at the ΔSCF level. In the ΔSCF method, the initial (*Ψ*_*i*_) and final (*Ψ*_*f*_) *N*-electron wave functions entering the dipole matrix element (computed in the length gauge of the dipole operator)
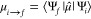
4are Slater determinants constructed from Kohn–Sham molecular orbitals (MOs) obtained with the SCF procedure relative to the ground state (GS) and excited state occupation numbers, respectively. The GS MOs are obtained from a spin-restricted calculation, while the excited-state MOs are calculated within a spin-polarized scheme with *N*_*α*_ – *N*_*β*_ = 0, where *N*_*α*_ and *N*_*β*_ denote the number of spin-up and spin-down electrons, respectively. In the specification of the occupation numbers, we removed a β electron from the HOMO. Singlet excitation energies are obtained according to the spin-purification formula.^45^ Denoting with *S*_*fi*_ the overlap matrix between the two sets of occupied MOs, (*S*_*fi*_)_*λμ*_ = ⟨φ_λ_^*f*^|φ_μ_^*i*^ ⟩, *μ*_*i*__→ *f*_, of eq 4 can be written as
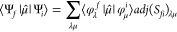
5in terms of dipole matrix elements between the two sets of MOs and the adjugate of *S*_*fi*_ (i.e., the transpose of its cofactor matrix). When ⟨*Ψ*_*f*_|*Ψ*_*i*_⟩ = *det*(*S*_*fi*_) ≠ 0, eq 5 reduces to
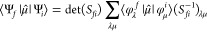
6

Since *Ψ*_*i*_ and *Ψ*_*f*_ do not need to be orthogonal, as they are the wave functions of fictitious systems, their use in the calculation of transition properties must be carefully justified. We always checked that, when not dictated by symmetry consideration, the overlap of the initial and final wave functions, ⟨*Ψ*_*f*_|*Ψ*_*i*_⟩, is actually very small (see Tables S18 and S19 of the Supporting Information). Moreover we always enforced the origin independence of the transition matrix elements by adding the dipole of the nuclear charges, weighted by the overlap ⟨*Ψ*_*f*_|*Ψ*_*i*_⟩. Even if this procedure does not eliminate the transition charge, results of a recent study^38^ on an extensive set of medium-size molecules indicate that this simple correction gives dipole moments nearly identical with those obtained by enforcing exact orthogonality of *Ψ*_*i*_ and *Ψ*_*f*_.

The equilibrium structures of the systems investigated, reported in Figure 2, have been optimized at the DFT level by using the PBE0^46−48^ xc functional and the triple ζ polarized (TZP) basis set of Slater type orbitals (STOs) from the ADF database. During the geometry optimization we did not impose any symmetry constraints. For both TDDFT and ΔSCF calculations, excitation energies and oscillator strengths have been calculated for the following classes of xc potentials: LDA VWN,^49^ GGA LB94,^50^ PBE,^46,47^ BLYP,^51−53^ PW86x,^54^ hybrid B3LYP,^52,55,56^ PBE0,^48,57^ BHandH,^58^ the meta-hybrid M06-2x,^59^ and the range-separated hybrid (RSH) CAM-B3LYP.^60^ In addition, two more recent range-separated hybrid functionals with the correct asymptotic potential, namely ωPBEh^61^ and ωB97x,^62^ have been tested as well. However, since they provide results that are of similar accuracy of CAM-B3LYP, we only include them in the Supporting Information. In Table S1, we compare, for TDDFT, the accuracy of the three range separated hybrid functionals in predicting the first vertical excitation energy for all the systems included in the benchmark study. As it appears from the statistical analysis reported in Table S1, ωPBEh and ωB97x RSH potentials provide results that are not more accurate than CAM-B3LYP. Furthermore, for BODIPY, we also optimally tuned^63,64^ two different long-range corrected potentials making use of the PLAMS Python library of ADF, namely LCY-PBE,^65^ and CAMY-B3LYP.^66^ However, the results for the first vertical excitation energy (CAMY-B3LYP, 3.153 eV, γ_opt_ = 0.55; LCY-PBE, 3.128 eV, γ_opt_ = 0.35) are comparable to those obtained with the three RSH potentials without optimal tuning. Optimal tuning the range separation of RSH potentials is therefore not further pursued in the present work. We also tested the model potential SAOP,^67,68^ which, together with LB94, should afford excitation energies somewhat more accurate than the standard GGA potentials.

## III Results and Discussion

The set of molecules considered in the present work are those reported in the works of refs (39 and 40). Except for molecule **4** (see Figure 2), for which the π → π* excitation shows a partial CT character, for all other systems, the transition has a local excitation character (see Supporting Information for B3LYP and PBE0 HOMO and LUMO MOs plots for systems **1**–**16**). For all xc functionals investigated in ref (39), it was shown that TDDFT cannot provide accurate results for the HOMO → LUMO vertical excitation (with positive deviations from experimental data greater than 0.3 eV), unless properly rescaled (for range-separated functionals). Moreover, the discrepancies with respect to experimental values could not be attributed to the neglect of solvent effects, which is estimated to be modest for all investigated systems. For correlated wave function methods, it was found that local CC2 (LCC2) and the DPLNO-STEOM-CCSD methods^69^ were suitable for the computation of vertical HOMO→ LUMO excitation energies, the former method being able to afford a high linear correlation with the experimental measurements.^40^ It is interesting to analyze, for the first singlet excited state calculated at the TDDFT level, the eigenvector of the Ω matrix, which is reported for **1** in Table 1 and for all other systems in Tables S2–S17 of the Supporting Information. In Table 1, we also report, for each xc potential used, the ΔSCF and TDDFT excitation energies and their signed error compared to the experimental value. For completeness, when available, also the CASPT2/cc-pVDZ vertical excitation values reported in ref (39) are reported in all tables. Focusing for the moment our attention on **1**, an analysis of the Ω matrix first eigenvector reveals that the transition does not correspond to a pure HOMO→ LUMO excitation and that the multideterminant character of the excitation strongly depends on the fraction of exact exchange included in the xc potential. While for all functionals the eigenvector can be described as a linear combination of HOMO → LUMO and HOMO–1 → LUMO single-particle excitations, the weight of the former increases roughly with the fraction of Hartree–Fock (HF) exchange in the potential, and it is a maximum for BHandH (50% of HF exchange) and for the meta-hybrid M06-2X potential (54% of HF exchange included). This trend is also reflected in the computed oscillator strength for the transition. All TDDFT functionals overestimate the excitation energy, and the disagreement with the experiment is larger for the hybrid/meta-hybrid and range-separated potentials. The trend on the signed error is different for the ΔSCF results (see also Figure S1 of the Supporting Information). For LDA GGA and model xc potentials the error is negative, and it progressively changes sign as the fraction of HF exchange included in the potential increases. The error is very small in magnitude for PBE0 and B3LYP, and it becomes larger for the range-separated potentials and M06-2X. On the other hand, the computed oscillator strength of the transition is less dependent on the particular xc potential used. The fact that the linear-response TDDFT shows a strong dependency of predicted excitation energies and oscillator strengths on the xc functional used can be traced back to the interplay between the Hartree and xc terms of the coupling matrix of eq 3. It is also interesting to note that the multideterminantal nature of the first excited state (S_1_) was clearly revealed in the analysis of the CI eigenvector of the CASSCF/cc-pVDZ results reported in ref (39). Also, at the CASSCF level, S_1_ can roughly be described as a linear combination of HOMO → LUMO and HOMO–1 → LUMO excitations, but the coefficients of doubly excited determinants are not negligible, both in the excited state and in the ground state. Even if the CASSCF wave function cannot afford an accurate description of the excited state wave function, it is clear that the importance of doubly excited states can be used to explain the poor performance of TDDFT in this class of systems. We note here that the effect of double and higher excitations can at least be partly described at the ΔSCF level, where excited state orbitals are self-consistently determined and can be used in principle as a better reference determinant for the excited state than single excitations built from the ground state reference KS determinant. The fact that orbital relaxation cannot be properly described only by single excitations out of the KS reference wave function can explain the higher accuracy, compared to TDDFT, of the excitation energies and oscillator strengths computed at the ΔSCF level, the latter being, for every system studied, quite close to the CASPT2 estimates (see also Tables S2–S17 of the Supporting Information). By inspecting the signed error of TDDFT and ΔSCF as a function of the xc potential for the other systems (Figures S2–S17 of the Supporting Information), some interesting trends can be identified. For the majority of the systems, an analysis of the errors similar to that of **1** can be made: TDDFT overestimates the transition energy for all xc potentials, while ΔSCF in combination with LDA and GGA xc potentials underestimates the transition energy. As the fraction of exact exchange included in the xc potential increases, the ΔSCF error becomes positive. This is however not a general behavior: at the ΔSCF level, the transition energy of the lowest energy excitation is overestimated for **5**, and in some systems, TDDFT is seen to outperform ΔSCF when used in combination with LDA and GGA potentials. In this respect, we should also note that CASPT2 estimates from ref (39) were used in instances where the experimental data were not available (systems **2**, **3**, **5**_**H**_, and **9**), and this can affect the comparison between TDDFT and ΔSCF performances. The accuracy of both ΔSCF and TDDFT methods is rather poor in **4**, and this is due to the partial CT character of the lowest energy transition in this system.^39,40^ It is anyway apparent that, with the exception of **4**, the ΔSCF method, when used with PBE0 and B3LYP xc potentials, is able to give results in remarkable good agreement with both the experiment and the CASPT2/cc-pVDZ estimates from ref (39). This last observation is put on a quantitative basis by performing a statistical analysis of the results, reported in Table 2 and Table 3 for TDDFT and ΔSCF respectively. In the tables, we perform the analysis both by including and by excluding system **4** from the set of molecules. From an inspection of Table 2, where the TDDFT results are reported, we note that irrespective of the choice of the xc potential, the mean absolute error (MAE) is quite large, ranging from values greater than 0.3 for LDA and GGA potentials to about 0.5 for xc potentials with some fraction of exact exchange included. The correlation (*R*^2^) between theoretical estimates and the experiment is also generally rather poor, but unlike the trend observed in the MAE, it is better for hybrid/meta-hybrid and range-separated potentials compared to LDA and GGA potentials. When **4** is excluded from the set, the statistics generally improve. In particular, the correlation reaches high values for M06-2X and CAM-B3LYP potentials (the *R*^2^ index also generally gets closer to the ideal value of 1.0 with increasing fraction of exact exchange). Therefore, even if the MAE is still large, in the interval between 0.3 to 0.5 eV, one can argue that if properly scaled TDDFT results with M06-2X or range separated xc potentials (see also Table S1 of the Supporting Information) can be used for a rough estimate of vertical excitation energies in similar systems. These observations agree with those reported in ref (27) about the performance of the M06-2X potential in predicting vertical excitation energies of BODIPY derivatives.

**Table 1:** ΔSCF and TDDFT Vertical Excitation Energies and Oscillator Strengths for the HOMO → LUMO Transition of **1**, for a Selection of DFT xc Potentials^a^

	ΔSCF		TDDFT		
XC	ε (eV)	*f*	ε (eV)	*f*	dominant excitations
BLYP	2.121 (−0.339)	0.48	3.003 (0.543)	0.16	0.49 H → L + 0.50 (H–1) → L
PBE	2.141 (−0.319)	0.48	3.017 (0.557)	0.15	0.48 H → L + 0.51 (H–1) → L
PW86X	2.123 (−0.337)	0.48	3.003 (0.543)	0.15	0.48 H → L + 0.51 (H–1) → L
B3LYP	2.414 (−0.046)	0.51	3.151 (0.691)	0.40	0.84 H → L + 0.15 (H–1) → L
PBE0	2.482 (0.022)	0.51	3.186 (0.726)	0.44	0.87 H → L + 0.11 (H–1) → L
CAM-B3LYP	2.894 (0.434)	0.54	3.137 (0.677)	0.52	0.94 H → L + 0.037 (H–1) → L
wPBEh	2.950 (0.490)	0.51	3.167 (0.707)	0.51	0.94 H → L + 0.039 (H–1) → L
wB97x	3.242 (0.782)	0.56	3.124 (0.664)	0.52	0.95 H → L + 0.021 (H–1) → L
VWN	2.184 (−0.276)	0.50	2.991 (0.531)	0.15	0.48 H → L + 0.51 (H–1) → L
SAOP	2.178 (−0.283)	0.49	3.058 (0.598)	0.23	0.61 H → L + 0.37 (H–1) → L
LB94	2.159 (−0.302)	0.49	2.93 (0.467)	0.18	0.56 H → L + 0.43 (H–1) → L
BHandH	2.855 (0.395)	0.55	3.189 (0.729)	0.54	0.95 H → L + 0.03 (H–1) → L
M06-2X	3.014 (0.554)	0.59	3.036 (0.576)	0.50	0.95 H → L + 0.03 (H–1) → L

a The differences with respect to the experimental value of 2.46 eV are also reported in parentheses. The CASPT2/cc-pVDZ value of the vertical excitation energy is 2.538 eV.^39^ For TDDFT, the eigenvector corresponding to the lowest energy root is also reported (H, HOMO; L, LUMO).

**Table 2:** TDDFT Data for the Lowest Vertical Energy Transition in the Entire Molecular Dataset Considered in This Work, together with a Statistical Analysis of the Results^a^

	LDA	model		GGA			hybrid			meta-hybrid	RSH
XC type	VWN	SAOP	LB94	BLYP	PBE	PW86X	B3LYP	PBE0	BHandH	M06-2X	CAM-B3LYP
**1**	2.991	3.058	2.927	3.003	3.017	3.003	3.151	3.186	3.189	3.036	3.137
**2**	2.710	2.758	2.643	2.728	2.740	2.725	2.888	2.920	2.894	2.765	2.851
**3**	2.789	2.864	2.659	2.796	2.816	2.803	3.216	3.330	3.672	3.675	3.637
**4**	2.835	2.891	2.711	2.835	2.851	2.841	3.277	3.393	3.772	3.562	3.748
**5**	3.627	3.664	3.484	3.654	3.675	3.659	3.876	3.952	4.129	4.043	4.093
**5_H_**	4.063	4.075	3.903	4.059	4.088	4.071	4.267	4.344	4.524	4.412	4.467
**6**	3.286	3.382	3.237	3.290	3.310	3.293	3.595	3.677	3.880	3.723	3.799
**7**	2.987	3.055	2.914	2.994	3.008	2.997	3.127	3.165	3.241	3.075	3.178
**8**	3.008	3.097	2.946	3.021	3.032	3.021	3.267	3.333	3.543	3.373	3.475
**9**	2.842	2.886	2.745	2.849	2.863	2.851	2.962	2.988	3.014	2.869	2.963
**10**	3.236	3.281	3.129	3.237	3.259	3.243	3.418	3.477	3.609	3.462	3.545
**11**	2.380	2.478	2.332	2.422	2.424	2.415	2.625	2.658	2.684	2.558	2.648
**12**	2.843	2.922	2.751	2.851	2.866	2.852	3.173	3.256	3.496	3.343	3.428
**13**	2.751	2.777	2.635	2.760	2.774	2.765	2.921	2.950	2.985	2.822	2.938
**14**	2.937	3.005	2.888	2.944	2.959	2.945	3.092	3.131	3.167	3.017	3.115
**15**	2.689	2.788	2.633	2.728	2.728	2.721	2.929	2.965	3.0185	2.856	2.968
**16**	2.692	2.757	2.633	2.719	2.726	2.732	2.845	2.875	2.900	2.758	2.859
MAE	0.359	0.401	0.342	0.368	0.373	0.368	0.471	0.509	0.589	0.474	0.541
MAE^b^	0.297	0.345	0.272	0.308	0.313	0.307	0.444	0.492	0.601	0.466	0.548
max AE	1.340	1.284	1.464	1.340	1.324	1.334	0.898	0.782	0.814	0.664	0.762
max AE^b^	0.584	0.652	0.600	0.591	0.606	0.592	0.739	0.778	0.814	0.664	0.762
min AE	0.013	0.048	0.004	0.026	0.037	0.026	0.043	0.071	0.386	0.274	0.329
min AE^b^	0.013	0.048	0.004	0.026	0.037	0.026	0.043	0.071	0.386	0.274	0.329
SD	0.295	0.278	0.320	0.295	0.293	0.294	0.214	0.191	0.127	0.104	0.116
SD^b^	0.169	0.174	0.159	0.172	0.177	0.173	0.191	0.183	0.122	0.101	0.116
R^2^	0.495	0.494	0.447	0.487	0.491	0.491	0.687	0.728	0.843	0.824	0.858
R^2^ b	0.797	0.805	0.761	0.798	0.801	0.800	0.908	0.925	0.958	0.970	0.965

a Reported are the mean absolute error (MAE), maximum and minimum AE (max AE and min AE, respectively), the standard deviation (SD), and the correlation coefficient (*R*^2^) between theoretical vertical excitation energies and experimental values. Energies in eV.

b Statistics obtained by removing molecule **4** from the data set.

**Table 3:** ΔSCF Data for the Lowest Vertical Energy Transition in the Entire Molecular Dataset Considered in This Work, together with a Statistical Analysis of the Results^a^

	LDA	model		GGA			hybrid			meta-hybrid	RSH
xc	VWN	SAOP	LB94	BLYP	PBE	PW86X	B3LYP	PBE0	BHandH	M06-2X	CAM-B3LYP
**1**	2.184	2.178	2.159	2.121	2.141	2.123	2.414	2.482	2.855	3.036	2.894
**2**	1.921	1.957	1.927	1.885	1.912	1.901	2.200	2.288	2.639	2.765	2.711
**3**	2.943	2.972	2.935	2.892	2.915	2.893	3.135	3.196	3.487	3.675	3.479
**4**	3.073	3.113	3.066	3.023	3.042	3.023	3.265	3.319	3.599	3.562	3.592
**5**	3.209	3.339	3.237	3.197	3.222	3.201	3.525	3.623	3.989	4.043	3.974
**5_H_**	3.596	3.685	3.601	3.555	3.588	3.566	3.891	3.996	4.387	4.412	4.338
**6**	2.824	2.848	2.803	2.755	2.773	2.748	3.048	3.101	3.444	3.723	3.472
**7**	2.115	2.176	2.123	2.097	2.108	2.098	2.485	2.576	3.051	3.075	-
**8**	2.321	2.4206	2.359	2.329	2.338	2.329	2.807	2.932	3.503	3.373	3.413
**9**	1.917	2.007	1.946	1.919	1.934	1.929	2.311	2.415	2.868	2.869	-
**10**	2.702	2.684	2.653	2.615	2.643	2.621	2.847	2.910	3.223	3.462	3.255
**11**	1.746	1.799	1.764	1.737	1.751	1.744	2.055	2.133	2.508	2.558	2.548
**12**	2.509	2.526	2.467	2.438	2.449	2.426	2.702	2.745	3.086	3.343	3.087
**13**	2.088	2.080	2.055	2.030	2.042	2.029	2.313	2.371	2.741	2.822	2.784
**14**	2.236	2.220	-	2.163	2.185	2.165	2.412	2.476	2.806	3.017	-
**15**	2.080	2.095	2.063	2.032	2.043	2.030	2.323	2.384	2.755	2.856	-
**16**	1.968	1.990	1.956	1.923	1.934	1.921	2.203	2.259	2.611	2.758	2.662
MAE	0.416	0.377	0.440	0.459	0.440	0.457	0.158	0.101	0.364	0.497	0.362
MAE^b^	0.374	0.335	0.396	0.416	0.397	0.413	0.111	0.054	0.351	0.494	0.343
max AE	1.102	1.062	1.109	1.152	1.133	1.152	0.910	0.857	0.576	0.624	0.583
max AE^b^	0.674	0.5745	0.636	0.666	0.657	0.666	0.247	0.142	0.508	0.624	0.459
min AE	0.117	0.133	0.288	0.190	0.168	0.188	0.046	0.0072	0.228	0.328	0.200
min AE^b^	0.117	0.133	0.288	0.190	0.168	0.188	0.046	0.0072	0.228	0.328	0.200
SD	0.217	0.197	0.199	0.206	0.206	0.205	0.196	0.192	0.093	0.072	0.102
SD^b^	0.137	0.101	0.102	0.114	0.115	0.113	0.057	0.037	0.079	0.073	0.083
R^2^	0.893	0.911	0.914	0.909	0.907	0.910	0.918	0.914	0.879	0.850	0.875
R^2^ b	0.939	0.967	0.967	0.960	0.9583	0.961	0.990	0.993	0.998	0.991	0.992

a Reported are the mean absolute error (MAE), maximum and minimum AE (max AE and min AE, respectively), the standard deviation (SD), and the correlation coefficient (*R*^2^) between theoretical vertical excitation energies and experimental values. Energies in eV.

b Statistics obtained by removing molecule **4** from the data set.

**Figure 3:**
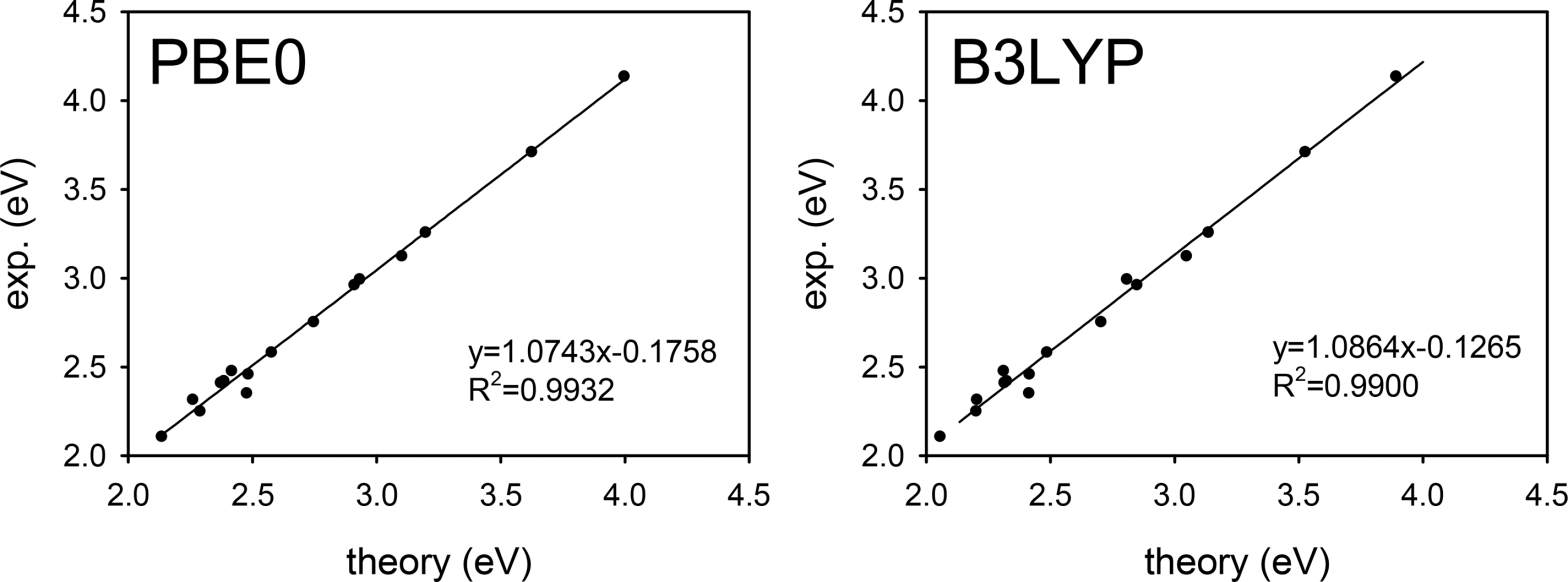
Comparison between ΔSCF calculated vertical excitation energies and experimental excitation energies of the set of molecules (**4** is excluded) investigated in this work. Left panel: PBE0 xc potential. Right panel: B3LYP xc potential.

From an analysis of the ΔSCF results (see Table 3), the general better accuracy of this method compared to TDDFT becomes quite clear. In particular, when ΔSCF is combined with xc potentials, which include a small fraction of exact exchange from HF theory (B3LYP with 20% and PBE0 with 25% respectively), in addition to relatively small MAE and standard deviation, an almost perfect linear correlation with the experimental data is obtained. (see Figure 3) Notably, the correlation is high (*R*^2^ greater than 0.9) irrespective of the xc potential, at variance with the TDDFT results. We could say that the accuracy afforded by the ΔSCF method for this class of systems is similar to that which is usually expected from the application of TDDFT to the calculation of HOMO → LUMO transitions in organic molecules. This observation and the effect of the fraction of exact exchange included in the xc potential on the accuracy of the ΔSCF estimate of the lowest π → π* excitation parallel the observations made by Ziegler et al. when studying the first π → π* excitation of cyanine dyes.^70^ There, the optimal fraction of exact exchange to be included in the xc potential for obtaining quite accurate ΔSCF transition energies was found at roughly 50%, while from our results, we find an optimal value around 20–25% for the BODIPY and aza-BODIPY families.

**Figure 4:**
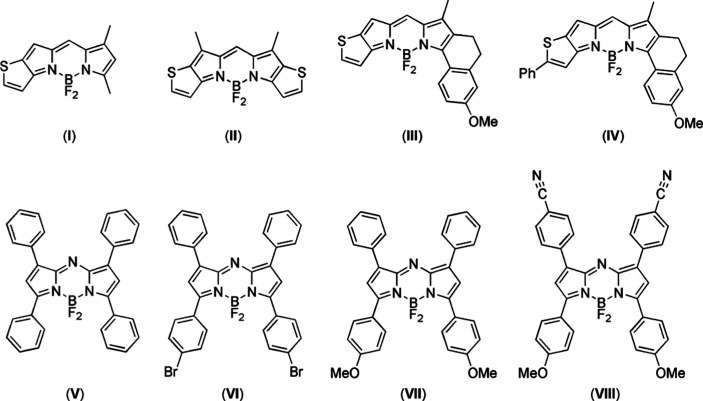
Conjugated BODIPYs and aza-BODIPYs considered in this work. Reprinted with permission from ref (40). Copyright 2021 Wiley.

Based on the results of the above benchmark, we decided to further test the accuracy of the ΔSCF method in conjunction with hybrid B3LYP and PBE0 xc potentials, by calculating the first vertical excitation energy of a set of eight BODIPYs/azaBODIPYs derivatives, whose structure is presented in Figure 4. The same set of conjugated BODIPYs and aza-BODIPYs were used to test the accuracy of LCC2 and RI-CC2 methods for predicting excitation energies for systems of increasing conjugation length.^39^ Results for the two xc potentials are reported in Table 4, together with their statistical analysis. A previous work^39^ has shown that typical errors of TDDFT for this set of conjugated systems are in the range 0.3–0.6 eV and that the errors associated with the LCC2/cc-pVTZ method could be as large as 0.4 eV, although characterized by a high *R*^2^ value when compared with the experimental values so that LCC2 rescaled energies based on a linear fit with the experimental values were in remarkably good agreement with the experiment.^40^ From the results reported in Table 4, we note that for both xc functionals an almost perfect correlation with the experimental data is obtained, comparable to that reported in ref (40) for LCC2 and the resolution-of-identity based CC2 methods, but with a much lower mean absolute error, the latter being largely below 0.1 eV when the PBE0 xc potential is used.

**Table 4:** ΔSCF PBE0/TZP and B3LYP/TZP Lowest Vertical Transition Energy for the Conjugated BODIPYs and aza-BODIPYs Shown in Figure 4, together with a Statistical Analysis of the Results^a^

	PBE0	B3LYP	expt
**I**	2.299	2.222	2.331^b^
**II**	2.184	2.103	2.206^c^
**III**	2.023	1.948	2.049^c^
**IV**	1.875	1.802	1.922^c^
**V**	1.867	1.777	1.907^d^^,^^e^
**VI**	1.836	1.744	1.884^f^
**VII**	1.747	1.662	1.802^d^^,^^e^^,^^g^
**VIII**	1.662	1.579	1.732^h^
mean AE	0.043	0.125	
min AE	0.022	0.101	
max AE	0.070	0.153	
SD	0.015	0.0180	
*R*^2^	0.9985	0.9979	

a Reported are the mean absolute error (MAE), maximum and minimum AE (max AE and min AE, respectively), the standard deviation (SD), and the correlation coefficient (*R*^2^) between theoretical vertical excitation energies and experimental values. Energies in eV.

b Reference (71).

c Reference (72).

d Reference (73).

e Reference (74).

f Reference (75).

g Reference (76).

h Reference (77).

## IV Conclusions

We computationally demonstrate that, provided a careful choice of xc potential is made, the ΔSCF method is able to deliver results of accuracy comparable to much more sophisticated wave function methods, at a fraction of the cost, for BODIPY and aza-BODIPY derivatives, which are challenging systems for the application of TDDFT. The poor performance of TDDFT in this class of systems seems to be related to the multideterminantal nature of the excited state with a character of double excitations, which are not included in standard TDDFT within the adiabatic approximation to the xc kernel. Based on the general greater accuracy afforded by the ΔSCF method, which includes naturally correlation effects due to electronic relaxation, we argue that the double excitation character of the first excited state of this class of systems is actually a reflection of relaxation effects following the electronic excitation.

Due to the spin-contamination of the HOMO → LUMO excited SCF wave function, it is not easy to quantify the amount of double-excitations in the excited state wave function. We attempted to perform such an analysis by calculating the overlap of the HOMO → LUMO excited SCF wave function with both the ground-state KS reference determinant and the manifold of single excitations out of the reference determinant. Results are reported in Tables S18 and S19 of the Supporting Information. As a consequence of spin-contamination, the weights of the HOMO → LUMO excited Slater determinant (which has by far the largest weight in the excited wave function) and of all single excitations are largely underestimated, and as a consequence, the weights of higher excitations are grossly overestimated. The importance of doubly and higher excited states in the accurate description of the lowest excited state of BODIPY/aza-BODIPY class of compounds has been evaluated on the basis of explicit wave function calculations by Momeni et al.^39^ Also, a recent benchmark paper using time-dependent double hybrid DFT on a similar class of systems^78^ support these findings.

In past computational studies dealing with the simulation of core–electron spectroscopies of boroxine-containing systems, we observed the superior accuracy of the ΔSCF method compared to both TDDFT and TP calculations with several xc potentials (ranging from LDA, GGA, hybrid, and RSH), so we are led to conclude that these electronic correlation (relaxation) effects should be quite widespread in B-containing systems and, therefore, not restricted to BODIPYs and aza-BODIPYs systems only. We could also argue that orbital-optimized excited state methods are to be preferred, compared to standard TDDFT within ALDA, for the description of electronically excited states of these systems, since orbital relaxation cannot be properly described only by single excitations out of the KS reference wave function. At variance with TDDFT, which can produce, with a single calculation, a large part (for medium-sized systems) of the absorption spectrum, the application of ΔSCF becomes challenging for the description of several excitations in the same molecule, since it is known to be plagued by convergence issues: in this work we could not converge the SCF cycle in some instances with the range-separated and LB94 xc potentials. We note however that excited state methods based on time-independent DFT have been put forward in recent years^79−82^ that are expected to be of similar accuracy to ΔSCF, and can be valid and cost-effective alternatives to TDDFT for problematic systems. These mean-field methods could represent an efficient computational strategy for a fast screening applied to a rational design of new chromophores with desired optical and spectroscopic properties.

## Supporting Information Available

Box charts showing, for systems **1**–**16**, the comparison between ΔSCF and TDDFT signed error of the first excitation energy with respect to the experimental value for the selection of xc potentials used; B3LYP and PBE0 HOMO and LUMO MO plots for systems **1**–**16**; comparison of the wPBEh, wB97x, and CAM-B3LYP lowest TDDFT vertical energy transition for systems **1**–**16**, together with a statistical analysis of the results; ΔSCF and TDDFT vertical excitation energies and the associated TDDFT eigenvector for systems **1**–**16**; and weight of the ground-state KS determinant and of the single-excitations out of the reference KS determinant in the PBE0 and B3LYP HOMO → LUMO excited SCF wave function for systems **1**–**16** (PDF)

## References

[ref1] Loudet A.; Burgess K. BODIPY Dyes and Their Derivatives: Syntheses and Spectroscopic Properties. Chem. Rev. 2007, 107 (11), 4891–4932. 10.1021/cr078381n. 17924696

[ref2] Kim K.; Jo C.; Easwaramoorthi S.; Sung J.; Kim D. H.; Churchill D. G. Crystallographic, Photophysical, NMR Spectroscopic and Reactivity Manifestations of the “8-Heteroaryl Effect” in 4,4-Difluoro-8-(C_4_H_3_X)-4-bora-3a,4a-diaza-s-indacene (X = O, S, Se) (BODIPY) Systems. Inorg. Chem. 2010, 49, 4881–4894. 10.1021/ic902467h. 20420417

[ref3] Poddar M.; Misra R. Recent Advances of BODIPY based Derivatives for optoelectronic applications. Coord. Chem. Rev. 2020, 421, 213462 10.1016/j.ccr.2020.213462.

[ref4] Nepomnyashchii A. B.; Bard A. J. Electrochemistry and Electrogenerated Chemiluminescence of BODIPY Dyes. Acc. Chem. Res. 2012, 45, 1844–1853. 10.1021/ar200278b. 22515199

[ref5] Chen D.; Zhong Z.; Ma Q.; Shao J.; Huang W.; Dong X. Aza-BODIPY-based Nanomedicines in Cancer Phototheranostics. ACS Appl. Mater. Interfaces 2020, 12, 26914–26925. 10.1021/acsami.0c05021. 32463220

[ref6] Yogo T.; Urano Y.; Ishitsuka Y.; Maniwa F.; Nagano T. Highly Efficient and Photostable Photosensitizer Based on BODIPY Chromophore. J. Am. Chem. Soc. 2005, 127, 12162–12163. 10.1021/ja0528533. 16131160

[ref7] Liu M.; Li C. Recent Advances in Activatable Organic Photosesitizers for Specific Photodynamics Therapy. ChemPlusChem. 2020, 85, 948–957. 10.1002/cplu.202000203. 32401421

[ref8] Batat P.; Cantuel M.; Jonusauskas G.; Scarpantonio L.; Palma A.; O’Shea D. F.; McClenaghan N. D. BF _2_ -Azadipyrromethenes: Probing the Excited-State Dynamics of a NIR Fluorophore and Photodynamic Therapy Agent. J. Phys. Chem. A 2011, 115 (48), 14034–14039. 10.1021/jp2077775. 22017189

[ref9] Kamkaew A.; Lim S. H.; Lee H. B.; Kiew L. V.; Chung L. Y.; Burgess K. BODIPY Dyes in Photodynamic Therapy. Chem. Soc. Rev. 2013, 42 (1), 77–88. 10.1039/C2CS35216H. 23014776 PMC3514588

[ref10] Boens N.; Leen V.; Dehaen W. Fluorescent Indicators Based on BODIPY. Chem. Soc. Rev. 2012, 41 (3), 1130–1172. 10.1039/C1CS15132K. 21796324

[ref11] Kolemen S.; Bozdemir O. A.; Cakmak Y.; Barin G.; Erten-Ela S.; Marszalek M.; Yum J.-H.; Zakeeruddin S. M.; Nazeeruddin M. K.; Gratzel M.; Akkaya E. U. Optimization of Distryryl-Dodipy Chromophores for Efficient Panchromatic Sensitization in Dye Sensitized Solar Cells. Chem. Sci. 2011, 2, 949–954. 10.1039/c0sc00649a.

[ref12] Rousseau T.; Cravino A.; Bura T.; Ulrich G.; Ziessel R.; Roncali J. BODIPY derivatives as Donor Materials for Bulk Heterojunction Solar Cells. Chem. Commun. 2009, 1673–1675. 10.1039/b822770e. 19294258

[ref13] Hattori S.; Ohkubo K.; Urano Y.; Sunahara H.; Nagano T.; Wada Y.; Tkachenko N. V.; Lemmetyinen H.; Fukuzumi S. J. Charge Separation in a Nonfluorescent Donor–Acceptor Dyad Derived from Boron Dipyrromethene Dye, Leading to Photocurrent Generation. J. Phys. Chem. B 2005, 109, 15368–15375. 10.1021/jp050952x. 16852949

[ref14] Yuan L.; Lin W.; Zheng K.; He L.; Huang W. Far-Red to near Infrared Analyte-Responsive Fluorescent Probes Based on Organic Fluorophore Platforms for Fluorescence Imaging. Chem. Soc. Rev. 2013, 42 (2), 622–661. 10.1039/C2CS35313J. 23093107

[ref15] Zhu L.; Wu W.; Zhu M.-Q.; Han J. J.; Hurst J. K.; Li A. D.Q. Reversibly Photoswitchable Dual-Color Fluorescent Nanoparticles as New Tools for Live-Cell Imaging. J. Am. Chem. Soc. 2007, 129, 3524–3526. 10.1021/ja068452k. 17335209 PMC2546355

[ref16] Runge E.; Gross E. K. U. Density-Functional Theory for Time-Dependent Systems. Phys. Rev. Lett. 1984, 52, 997 10.1103/PhysRevLett.52.997.

[ref17] Casida M. E. Time-Dependent Density Functional Response Theory for Molecules, In Recent Advances in Density Functional Methods; Chong D. P., Ed.; World Scientific: Singapore, 1995; pp 155–193.

[ref18] Hattig C.; Weigend F. CC2 excitation energy calculations on large molecules using the resolution of the identity approximation. J. Chem. Phys. 2000, 113, 5154–5161. 10.1063/1.1290013.

[ref19] Hattig C. Structure Optimizations for Excited States with Correlated Second-Order Methods. Adv. Quantum Chem. 2005, 50, 37–60. 10.1016/S0065-3276(05)50003-0.

[ref20] Christiansen O.; Koch H.; Jorgensen P. The second-order approximate coupled cluster singles and doubles model CC2. Chem. Phys. Lett. 1995, 243, 409–418. 10.1016/0009-2614(95)00841-Q.

[ref21] McDouall J. J.; Peasley K.; Robb M. A. A simple MC SCF perturbation theory: Orthogonal valence bond Møller-Plesset 2 (OVB MP2). Chem. Phys. Lett. 1988, 148, 183–189. 10.1016/0009-2614(88)80296-3.

[ref22] Helgaker T.; Jorgensen P.; Olsen J. Molecular Electronic-Structure Theory; John Wiley & Sons Inc.: 2013.

[ref23] Wen J.; Han B.; Havlas Z.; Michl J. An MS-CASPT2 Calculation of the Excited Electronic States of an Axial Difluoroborondipyrromethene (BODIPY) Dimer. J. Chem. Theory Comput. 2018, 14, 4291–4297. 10.1021/acs.jctc.8b00136. 29874458

[ref24] le Guennic B.; Maury O.; Jacquemin D. Aza-boron-dipyrromethene dyes: TD-DFT benchmarks, spectral analysis and design of original near-IR structures. Phys. Chem. Chem. Phys. 2012, 14, 157–164. 10.1039/C1CP22396H. 22068264

[ref25] Schlachter A.; Fleury A.; Tanner K.; Soldera A.; Habermeyer B.; Guilard R.; Harvey P. D. The TDDFT Excitation Energies of the BODIPYs; The DFT and TDDFT Challenge Continues. Molecules 2021, 26, 1780 10.3390/molecules26061780. 33810021 PMC8005089

[ref26] Charaf-Eddin A.; Le Guennic B.; Jacquemin D. Exited-state of BODIPY-cyanines: ultimate TD-DFT challenges?. RSC Adv. 2014, 4, 49449 10.1039/C4RA09494H.

[ref27] Chibani S.; Le Guennic B.; Charaf-Eddin A.; Laurent A. D.; Jacquemin D. Revisiting the optical signatures of BODIPY with ab-initio tools. Chem. Sci. 2013, 4, 1950–1963. 10.1039/c3sc22265a.

[ref28] Tozer J. D. Relationship between long-range charge-transfer excitation energy error and integer discontinuity in Kohn–Sham theory. J. Chem. Phys. 2003, 119, 12697–12699. 10.1063/1.1633756.

[ref29] Dreuw A.; Head-Gordon M. Failure of Time-Dependent Density Functional Theory for Long-Range Charge-Transfer Excited States: The Zincbacteriochlorin–Bacteriochlorin and Bacteriochlorophyll–Spheroidene Complexes. J. Am. Chem. Soc. 2004, 126, 4007–4016. 10.1021/ja039556n. 15038755

[ref30] Boulanger B.; Chibani S.; Le Guennic B.; Duchemin I.; Blasé X.; Jacquemin D. Combining the Bethe-Salpeter Formalism with Time-Dependent DFT excited-State Forces to Describe Optical Signatures: NBO Fluoroborates as Working Examples. J. Chem. Theory Comput. 2014, 10, 4548–4556. 10.1021/ct500552e. 26588148

[ref31] Head-Gordon M.; Maurice D.; Oumi M. A perturbative correction to restricted open shell configuration interaction with single substitutions for excited states of radicals. Chem. Phys. Lett. 1995, 246, 114–121. 10.1016/0009-2614(95)01111-L.

[ref32] Petrushenko I. K.; Petrushenko K. B. Effect of meso-substituents on the electronic transitions of BODIPY dyes: DFT and RI-CC2 study. Spectrohimica Acta Part A: Molecular and Biomolecular Spectroscopy 2015, 138, 623–627. 10.1016/j.saa.2014.12.005. 25541400

[ref33] Le Guennic B.; Jacquemin D. Taking Up the Cyanine Challenge with Quantum Tools. Acc. Chem. Res. 2015, 48, 530–537. 10.1021/ar500447q. 25710687 PMC4365665

[ref34] Toffoli D.; Bernes E.; Cossaro A.; Balducci G.; Stener M.; Mauri S.; Fronzoni G. Computational NEXAFS Characterization of Molecular Model Systems for 2D Boroxine Frameworks. Nanomaterials 2022, 12, 1610 10.3390/nano12091610. 35564319 PMC9100003

[ref35] Toffoli D.; Grazioli C.; Monti M.; Stener M.; Totani R.; Richter R.; Schio L.; Fronzoni G.; Cossaro A. Revealing the electronic properties of the B–B bond: the bis-catecholato diboron molecule. Phys. Chem. Chem. Phys. 2021, 23, 23517–23525. 10.1039/D1CP03428F. 34642728

[ref36] Toffoli D.; Ponzi A.; Bernes E.; de Simone M.; Grazioli C.; Coreno M.; Stredansky M.; Cossaro A.; Fronzoni G. Correlation effects in B1s core-excited states of boronic-acid derivatives: An experimental and computational study. J. Chem. Phys. 2019, 151, 134306 10.1063/1.5120175. 31594342

[ref37] Toffoli D.; Stredansky M.; Feng Z.; Balducci G.; Furlan S.; Stener M.; Ustunel H.; Cvetko D.; Kladnik G.; Morgante A.; et al. Electronic properties of the boroxine–gold interface: evidence of ultra-fast charge delocalization. Chem. Sci. 2017, 8, 3789–3798. 10.1039/C6SC05632F. 28580111 PMC5436552

[ref38] Bourne Worster S.; Feighan O.; Manby F. R. Reliable transition properties from excited-state mean-field calculations. J. Chem. Phys. 2021, 154, 124106 10.1063/5.0041233. 33810673

[ref39] Momeni M. R.; Brown A. Why do TD-DFT Excitation Energies of BODIPY/aza-BODIPY Families Largely Deviate from Experiment? Answers from Electron Correlated and Multireference Methods. J. Chem. Theory Comput. 2015, 11, 2619–2632. 10.1021/ct500775r. 26575559

[ref40] Feldt M.; Brown A. Assessment of local coupled cluster methods for excited states of BODIPY/Aza-BODIPY families. J. Comput. Chem. 2021, 42, 144–155. 10.1002/jcc.26442. 33103817

[ref41] Fonseca Guerra C.; Snijders J. G.; teVelde G.; Baerends E. J. Towards an Order-N DFT Method. Theor. Chem. Acc. 1998, 99, 391–403. 10.1007/s002140050353.

[ref42] Baerends E. J.; Ellis D. E.; Ros P. Self-Consistent Molecular Hartree-Fock-Slater Calculations I. The Computational Procedure. Chem. Phys. 1973, 2, 41–51. 10.1016/0301-0104(73)80059-X.

[ref43] te Velde G.; Bickelhaupt F. M.; Baerends E. J.; Fonseca Guerra C.; van Gisbergen S. J.A.; Snijders J. G.; Ziegler T. Chemistry with ADF. J. Comput. Chem. 2001, 22, 931–967. 10.1002/jcc.1056.

[ref44] Davidson E. R. The Iterative Calculation of a Few of the Lowest Eigenvalues and Corresponding Eigenvectors of Large Real-Symmetric Matrices. J. Comput. Phys. 1975, 17, 87–94. 10.1016/0021-9991(75)90065-0.

[ref45] Ziegler T.; Rauk A.; Baerends E. J. On the calculation of multiplet energies by the Hartree-Fock-Slater method. Theor. Chim. Acta 1977, 43, 261–271. 10.1007/BF00551551.

[ref46] Perdew J. P.; Burke K.; Ernzerhof M. Generalized Gradient Approximation Made Simple. Phys. Rev. Lett. 1996, 77, 3865–3868. 10.1103/PhysRevLett.77.3865. 10062328

[ref47] Perdew J. P.; Burke K.; Ernzerhof M. Generalized Gradient Approximation Made Simple. Phys. Rev. Lett. 1997, 78, 1396–1396. 10.1103/PhysRevLett.78.1396. 10062328

[ref48] Adamo C.; Barone V. Toward reliable density functional methods without adjustable parameters: The PBE0 model. J. Chem. Phys. 1999, 110, 6158–6169. 10.1063/1.478522.

[ref49] Vosko S. H.; Wilk L.; Nusair M. Accurate spin-dependent electron liquid correlation energies for local spin density calculations: a critical analysis. Can. J. Phys. 1980, 58, 1200–1211. 10.1139/p80-159.

[ref50] van Leeuwen R.; Baerends E. J. Exchange-correlation potential with correct asymptotic behavior. Phys. Rev. A 1994, 49, 2421–2431. 10.1103/PhysRevA.49.2421. 9910514

[ref51] Becke A. D. Density-functional exchange-energy approximation with correct asymptotic behavior. Phys. Rev. A 1988, 38, 3098–3100. 10.1103/PhysRevA.38.3098. 9900728

[ref52] Lee C.; Yang W.; Parr R. G. Development of the Colle-Salvetti correlation-energy formula into a functional of the electron density. Phys. Rev. B 1988, 37, 785–789. 10.1103/PhysRevB.37.785. 9944570

[ref53] Miehlich B.; Savin A.; Stoll H.; Preuss H. Results obtained with the correlation energy density functionals of Becke and Lee, Yang and Parr. Chem. Phys. Lett. 1989, 157, 200–206. 10.1016/0009-2614(89)87234-3.

[ref54] Perdew J. P.; Yue W. Accurate and simple density functional for the electronic exchange energy: generalized gradient approximation. Phys. Rev. B 1986, 33, 8800–8802. 10.1103/PhysRevB.33.8800. 9938293

[ref55] Becke A. D. Density-functional thermochemistry. III. The role of exact exchange. J. Chem. Phys. 1993, 98, 5648–5652. 10.1063/1.464913.

[ref56] Stephens P. J.; Devlin F. J.; Chabalowski C. F.; Frisch M. J. Ab Initio Calculation of Vibrational Absorption and Circular Dichroism Spectra Using Density Functional Force Fields. J. Phys. Chem. 1994, 98, 11623–11627. 10.1021/j100096a001.

[ref57] Ernzerhof M.; Scuseria G. Assessment of the Perdew-Burke-Ernzerhof exchange-correlation functional. J. Chem. Phys. 1999, 110, 5029–5036. 10.1063/1.478401. 15268348

[ref58] Becke A. D. A new mixing of Hartree-Fock and local density-functional theories. J. Chem. Phys. 1993, 98, 1372–1377. 10.1063/1.464304.

[ref59] Zhao Y.; Truhlar D. G. The M06 suite of density functionals for main group thermochemistry, thermochemical kinetics, noncovalent interactions, excited states, and transition elements: two new functionals and systematic testing of four M06-class functionals and 12 other functionals. Theor. Chem. Acc. 2008, 120, 215–241. 10.1007/s00214-007-0310-x.

[ref60] Yanai T.; Tew D. P.; Handy N. C. A new hybrid exchange–correlation functional using the Coulomb-attenuating method (CAM-B3LYP). Chem. Phys. Lett. 2004, 393, 51–57. 10.1016/j.cplett.2004.06.011.

[ref61] Rohrdanz M. A.; Martins K. M.; Herbert J. M. A long-range-corrected density functional that performs well for both ground-state properties and time-dependent density functional theory excitation energies, including charge-transfer excited states. J. Chem. Phys. 2009, 130, 054112 10.1063/1.3073302. 19206963

[ref62] Chai J.-D.; Head-Gordon M. Systematic optimization of long-range corrected hybrid density functionals. J. Chem. Phys. 2008, 128, 084106 10.1063/1.2834918. 18315032

[ref63] Kronik L.; Stein T.; Refaely-Abramson S.; Baer R. Excitation Gaps of Finite-Sized Systems from Optimally Tuned Range-Separated Hybrid Functionals. J. Chem. Theory Comput. 2012, 8, 1515–1531. 10.1021/ct2009363. 26593646

[ref64] Janesko B. G. Benchmarking time-dependent density functional theory prediction of emission spectra and CIE color: A rainbow of error. Int. J. Quantum Chem. 2022, 122, e26970 10.1002/qua.26970.

[ref65] Seth M.; Ziegler T. Range-Separated Exchange Functionals with Slater-Type Functions. J. Chem. Theory Comput. 2012, 8, 901–907. 10.1021/ct300006h. 26593352

[ref66] Akinaga Y.; Ten-no S. Range-separation by the Yukawa potential in long-range corrected density functional theory with Gaussian-type basis functions. Chem. Phys. Lett. 2008, 462, 348–351. 10.1016/j.cplett.2008.07.103.

[ref67] Schipper P. R. T.; Gritsenko O. V.; van Gisbergen S. J. A.; Baerends E. J. Molecular calculations of excitation energies and (hyper)polarizabilities with a statistical average of orbital model exchange-correlation potentials. J. Chem. Phys. 2000, 112, 1344–1352. 10.1063/1.480688.

[ref68] Gritsenko O. V.; Schipper P. R. T.; Baerends E. J. Approximation of the exchange-correlation Kohn-Sham potential with a statistical average of different orbital model potentials. Chem. Phys. Lett. 1999, 302, 199–207. 10.1016/S0009-2614(99)00128-1.

[ref69] Berraud-Pache R.; Neese F.; Bistoni G.; Izsák R. Unveiling the Photophysical Properties of Boron-dipyrromethene Dyes Using a New Accurate Excited State Coupled Cluster Method. J. Chem. Theory Comput. 2020, 16, 564–575. 10.1021/acs.jctc.9b00559. 31765141

[ref70] Zhekova H.; Krykunov M.; Autschbach J.; Ziegler T. Applications of Time Dependent and Time Independent Density Functional Theory to the First π to π* Transition in Cyanine Dyes. J. Chem. Theory Comput. 2014, 10, 3299–3307. 10.1021/ct500292c. 26588299

[ref71] Jiang X.-D.; Zhang H.; Zhang Y.; Zhao W. Development of non-symmetric thiophene-fused BODIPYs. Tetrahedron 2012, 68, 9795–9801. 10.1016/j.tet.2012.09.011.

[ref72] Tanaka K.; Yamane H.; Yoshii R.; Chujo Y. Efficient light absorbers based on thiophene-fused boron dipyrromethene (BODIPY) dyes. Bioorg. Med. Chem. 2013, 21, 2715–2719. 10.1016/j.bmc.2013.03.050. 23583692

[ref73] Killoran J.; Allen L.; Gallagher J. F.; Gallagher W. M.; O’Shea D. F. Synthesis of BF2 chelates of tetraarylazadipyrromethenes and evidence for their photodynamic therapeutic behavior. Chem. Commun. 2002, 1862–1863. 10.1039/B204317C. 12271646

[ref74] Bellier Q.; Pegaz S.; Aronica C.; Guennic B. L.; Andraud C.; Maury O. Near-Infrared Nitrofluorene Substitued Aza-Boron-dipyrromethenes Dyes. Org. Lett. 2011, 13, 22–25. 10.1021/ol102701v. 21128642

[ref75] Bouit P.-A.; Kamada K.; Feneyrou P.; Berginc G.; Toupet L.; Maury O.; Andraud C. Two-Photon Absorption-Related Properties of Functionalized BODIPY Dyes in the Infrared Range up to Telecommunication Wavelengths. Adv. Mater. 2009, 21, 1151–1154. 10.1002/adma.200801778.

[ref76] Gorman A.; Killoran J.; O’Shea C.; Kenna T.; Gallagher W. M.; O’Shea D. F. In vitro demonstration of the heavy-atom effect for photodynamic therapy. J. Am. Chem. Soc. 2004, 126, 10619–10631. 10.1021/ja047649e. 15327320

[ref77] Jiao L.; Wu Y.; Wang S.; Hu X.; Zhang P.; Yu C.; Cong K.; Meng Q.; Hao E.; Vicente M. G. H. Accessing Near-Infrared-Absorbing BF2-Azadipyrromethenes via a Push–Pull Effect. J. Org. Chem. 2014, 79, 1830–1835. 10.1021/jo402160b. 24476041 PMC4415601

[ref78] Helal W.; Alkhatib Q.; Gharaibeh M. Can time-dependent double hybrid density functionals accurately predict electronic excitation energies of BODIPY compounds?. Comp. Theor. Chem. 2022, 1207, 113531 10.1016/j.comptc.2021.113531.

[ref79] Ziegler T.; Seth M.; Krykunov M.; Autschbach J.; Wang F. On the relation between time-dependent and variational density functional theory approaches for the determination of excitation energies and transition moments. J. Chem. Phys. 2009, 130, 154102 10.1063/1.3114988. 19388731

[ref80] Ramos P.; Pavanello M. Low-lying excited states by constrained DFT. J. Chem. Phys. 2018, 148, 144103 10.1063/1.5018615. 29655334

[ref81] Hait D.; Head-Gordon M. Excited State Orbital Optimization via Minimizing the Square of the Gradient: General Approach and Application to Singly and Doubly Excited States via Density Functional Theory. J. Chem. Theory Comput. 2020, 16, 1699–1710. 10.1021/acs.jctc.9b01127. 32017554

[ref82] Evangelista F. A.; Shushkov P.; Tully J. C. Orthogonality Constrained Density Functional Theory for Electronic Excited States. J. Phys. Chem. A 2013, 117, 7378–7392. 10.1021/jp401323d. 23590595

