# Supplementary material for: Accurate Vertical Excitation Energies of BODIPY/Aza-BODIPY Derivatives from Excited-State Mean-Field Calculations
Source: J Phys Chem A. 2022 Sep 29;126(40):7137–46. doi: 10.1021/acs.jpca.2c04473 (PMC9574914; doi:10.1021/acs.jpca.2c04473)
Supplement: Supplementary file 1 — jp2c04473_si_001.pdf [file jp2c04473_si_001.pdf]

Supporting Information for: Accurate Vertical Excitation Energies of BODIPY/Aza-BODIPY Derivatives from Excited-State Mean-Field Calculations

Daniele Toffoli[†,‡]*, Matteo Quarin*[†]*, Giovanna Fronzoni*[†]*, and Mauro Stener*[†,‡] *

[†]Dipartimento di Scienze Chimiche e Farmaceutiche, Università degli Studi di Trieste, via L. Giorgieri 1, I-34127 Trieste, Italy

[‡]CNR-IOM, Istituto Officina dei Materiali, I-34149, Trieste, Italy

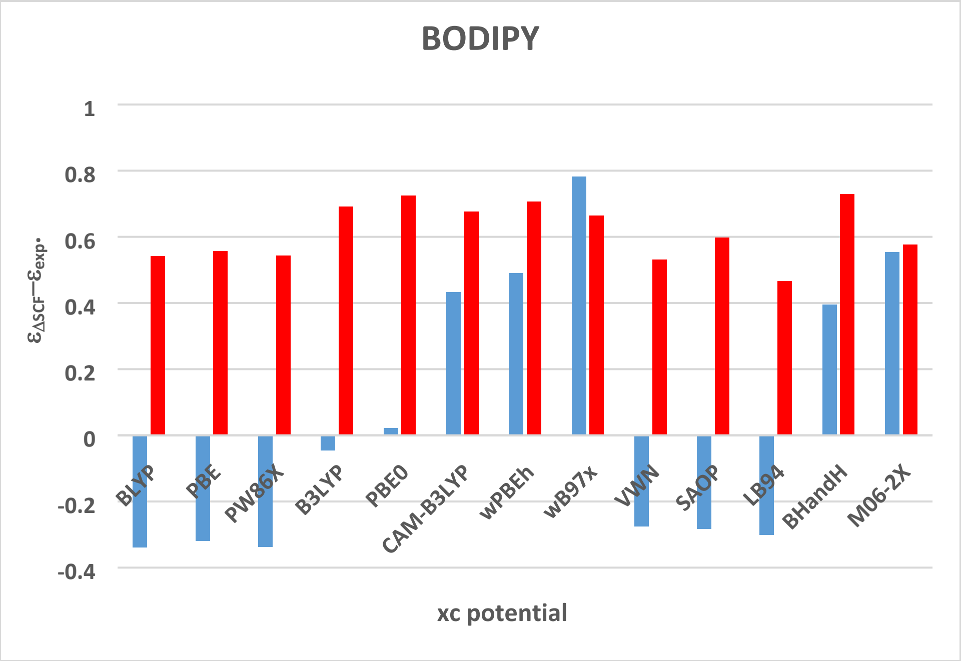

Figure S1. Box chart showing the comparison between ΔSCF (blue) and TDDFT (red) signed error of the first excitation energy with respect to the experimental value for the selection of DFT *xc* potentials for **1**.

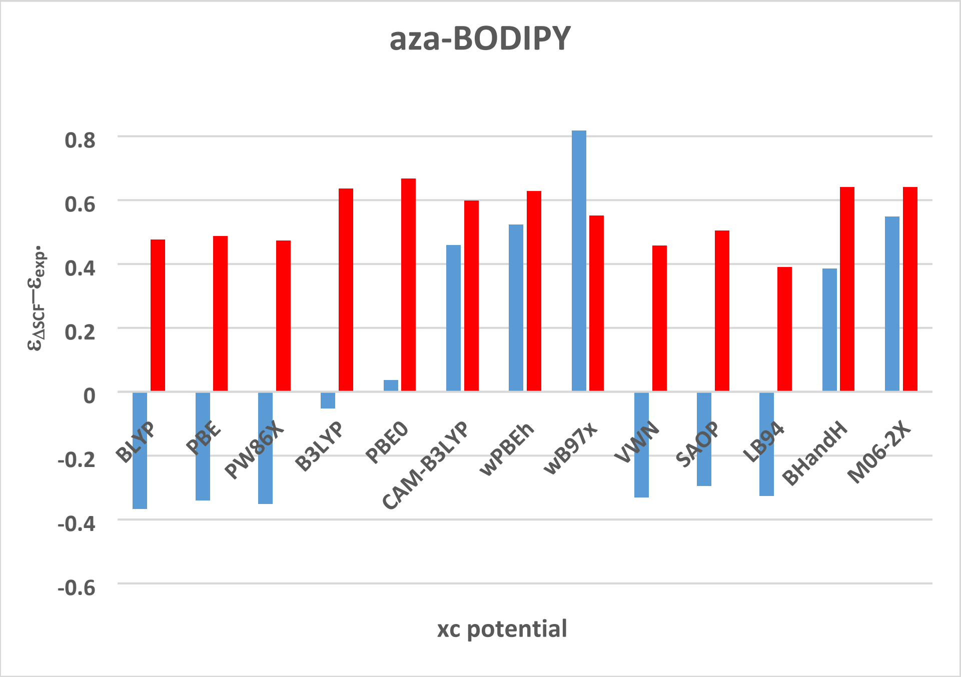

Figure S2. Box chart showing the comparison between ΔSCF (blue) and TDDFT (red) signed error of the first excitation energy with respect to the experimental value for the selection of DFT *xc* potentials for **2**.

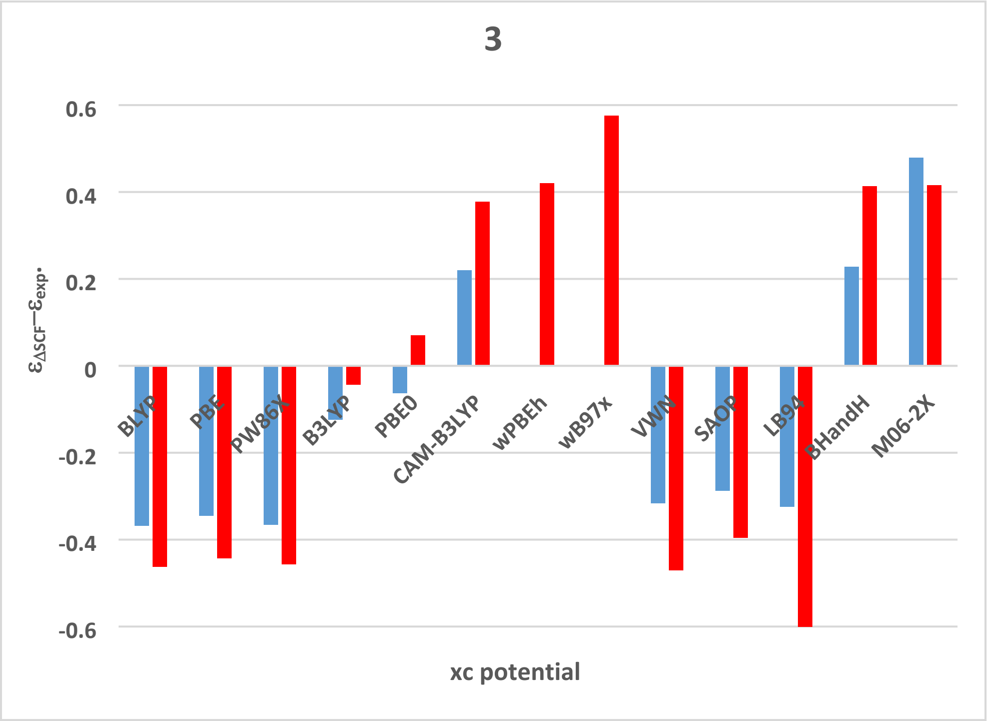

Figure S3. Box chart showing the comparison between ΔSCF (blue) and TDDFT (red) signed error of the first excitation energy with respect to the experimental value for the selection of DFT *xc* potentials for **3**.

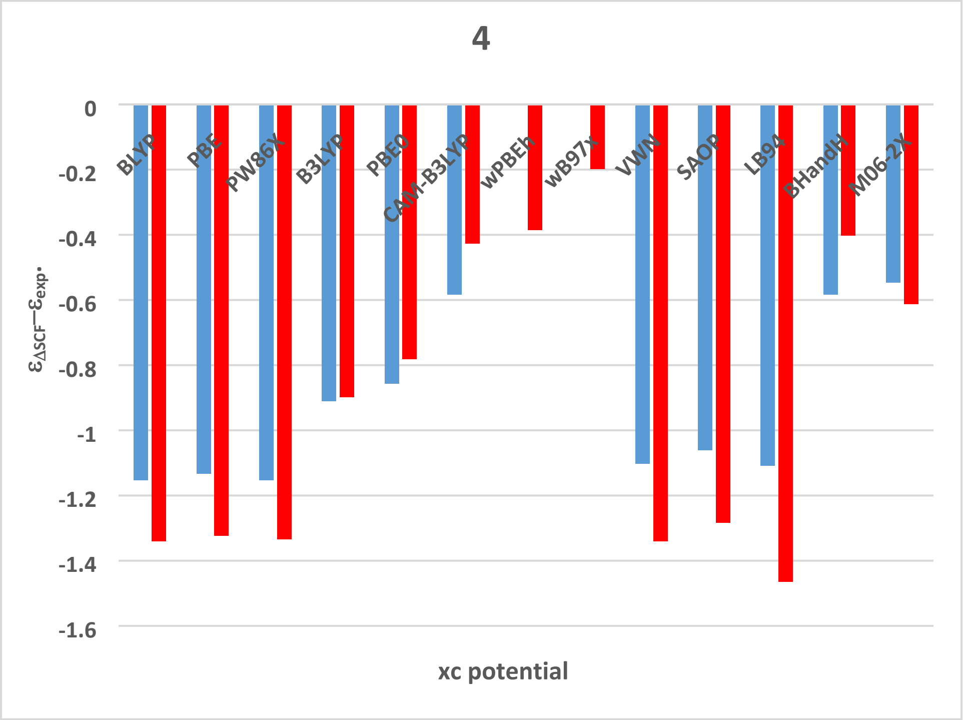

Figure S4. Box chart showing the comparison between ΔSCF (blue) and TDDFT (red) signed error of the first excitation energy with respect to the experimental value for the selection of DFT *xc* potentials for **4**.

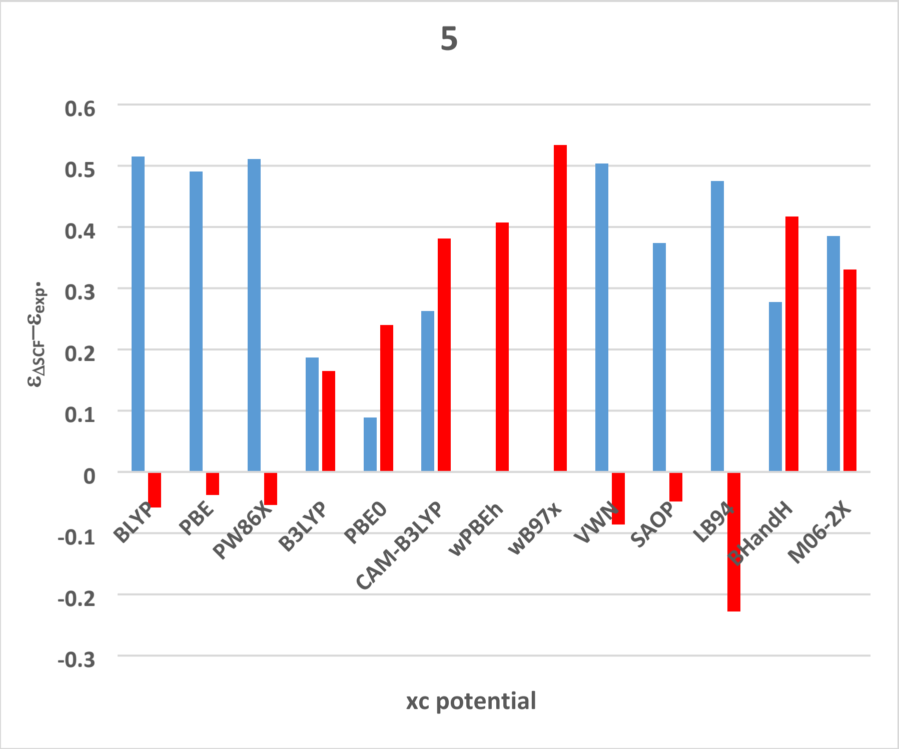

Figure S5. Box chart showing the comparison between ΔSCF (blue) and TDDFT (red) signed error of the first excitation energy with respect to the experimental value for the selection of DFT *xc* potentials for **5**.

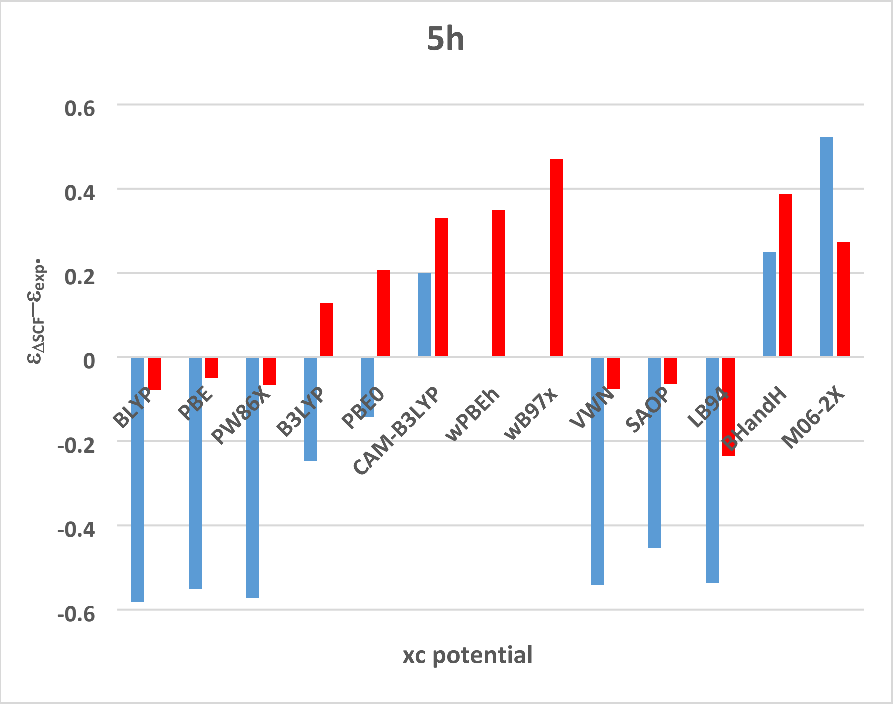

Figure S6. Box chart showing the comparison between ΔSCF (blue) and TDDFT (red) signed error of the first excitation energy with respect to the experimental value for the selection of DFT *xc* potentials for **5h**.

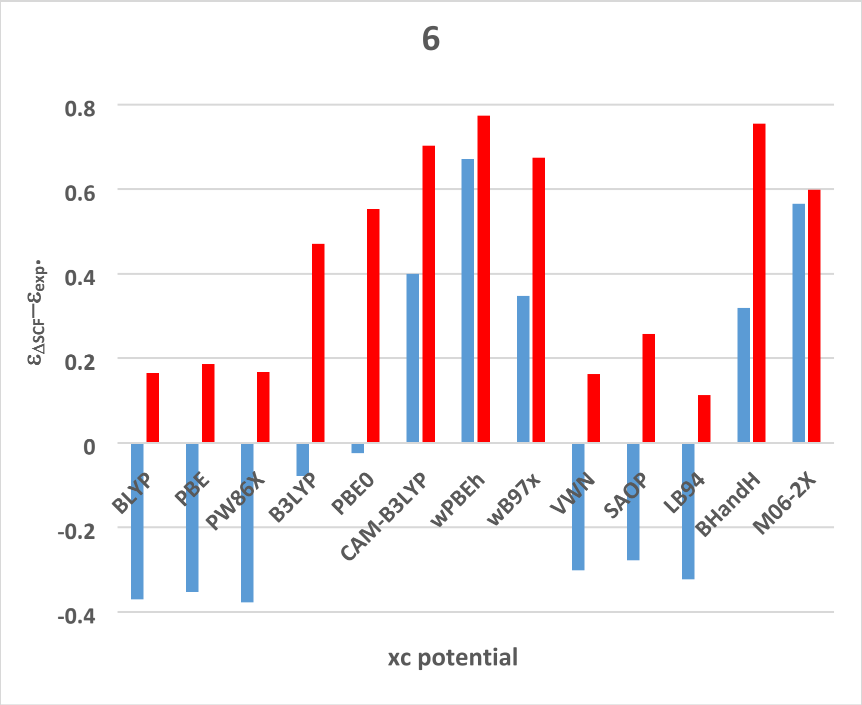

Figure S7. Box chart showing the comparison between ΔSCF (blue) and TDDFT (red) signed error of the first excitation energy with respect to the experimental value for the selection of DFT *xc* potentials for **6**.

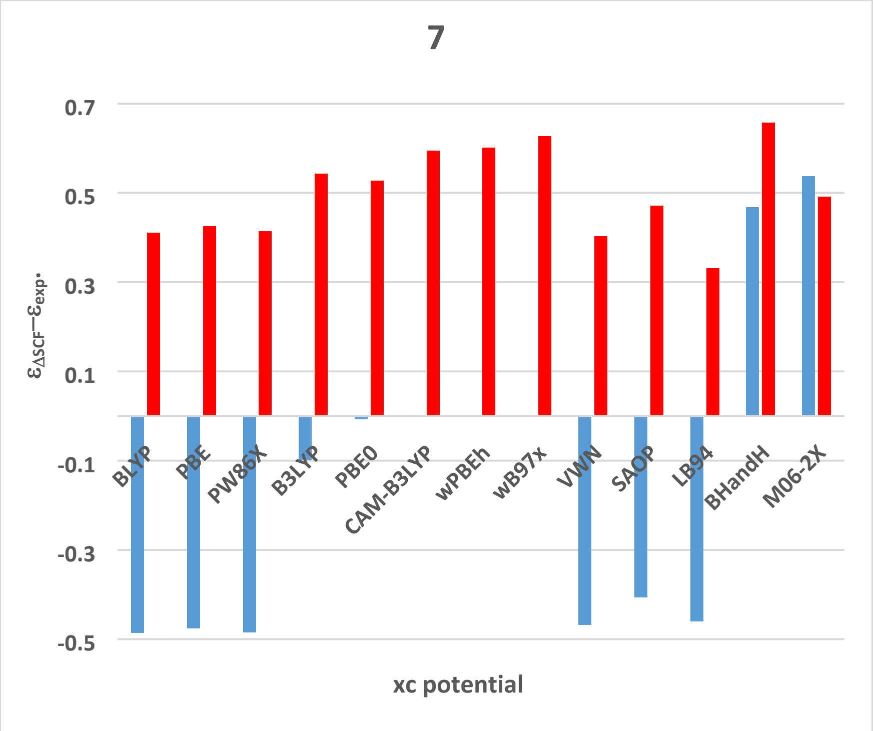

Figure S8. Box chart showing the comparison between ΔSCF (blue) and TDDFT (red) signed error of the first excitation energy with respect to the experimental value for the selection of DFT *xc* potentials for **7**.

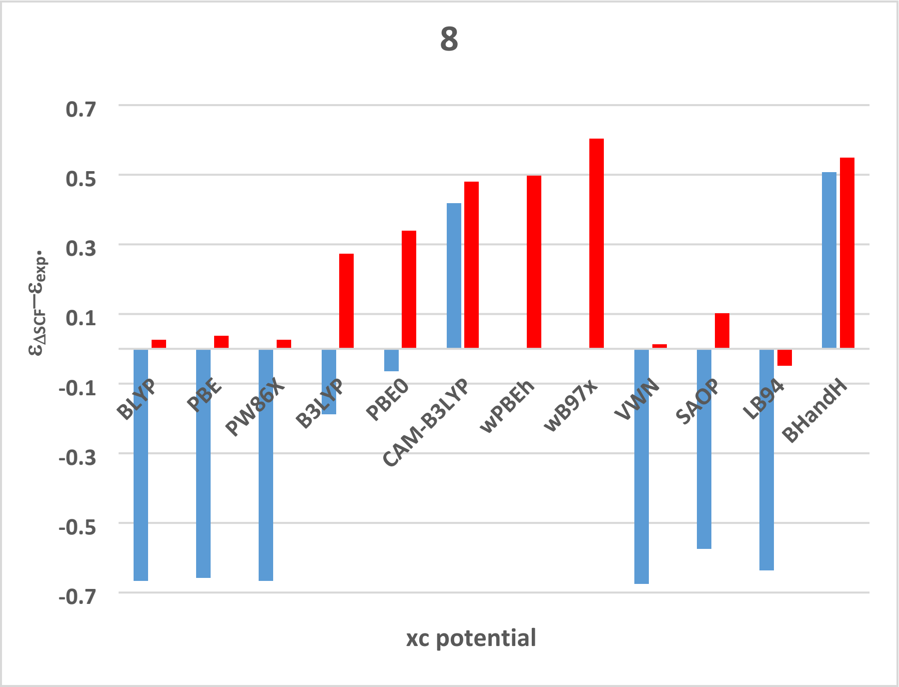

Figure S9. Box chart showing the comparison between ΔSCF (blue) and TDDFT (red) signed error of the first excitation energy with respect to the experimental value for the selection of DFT *xc* potentials for **8**.

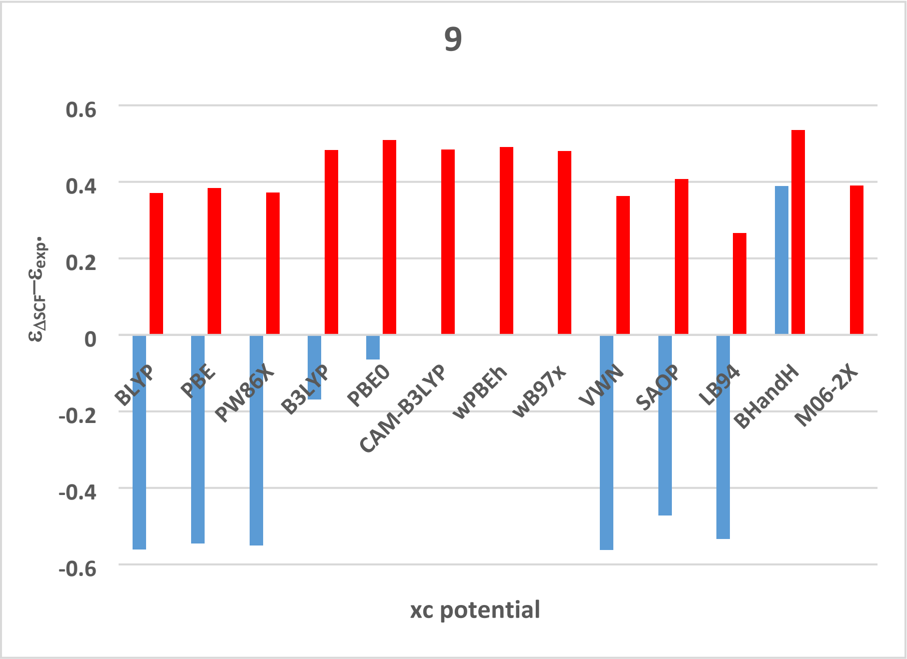

Figure S10. Box chart showing the comparison between ΔSCF (blue) and TDDFT (red) signed error of the first excitation energy with respect to the experimental value for the selection of DFT *xc* potentials for **9**.

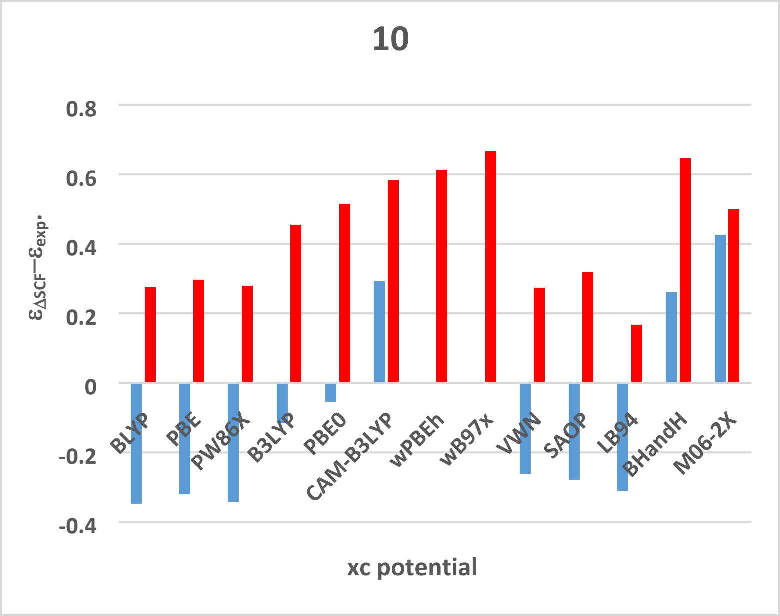

Figure S11. Box chart showing the comparison between ΔSCF (blue) and TDDFT (red) signed error of the first excitation energy with respect to the experimental value for the selection of DFT *xc* potentials for **10**.

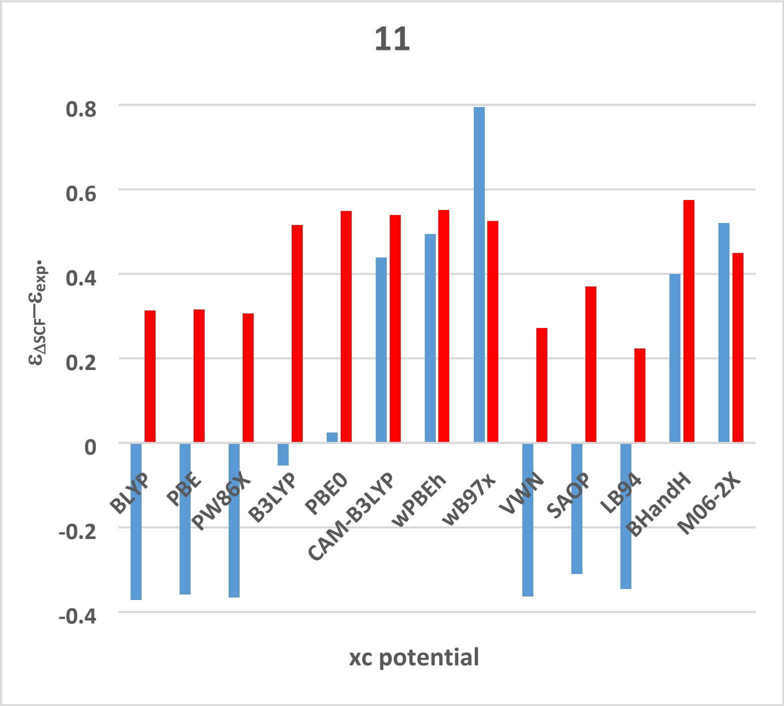

Figure S12. Box chart showing the comparison between ΔSCF (blue) and TDDFT (red) signed error of the first excitation energy with respect to the experimental value for the selection of DFT *xc* potentials for **11.**

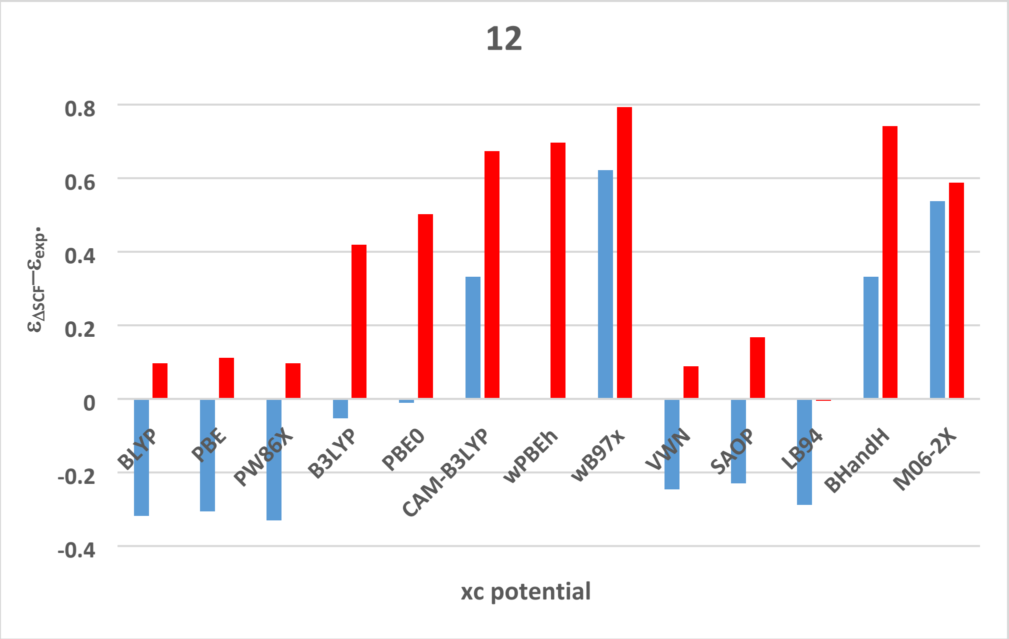

Figure S13. Box chart showing the comparison between ΔSCF (blue) and TDDFT (red) signed error of the first excitation energy with respect to the experimental value for the selection of DFT *xc* potentials for **12**.

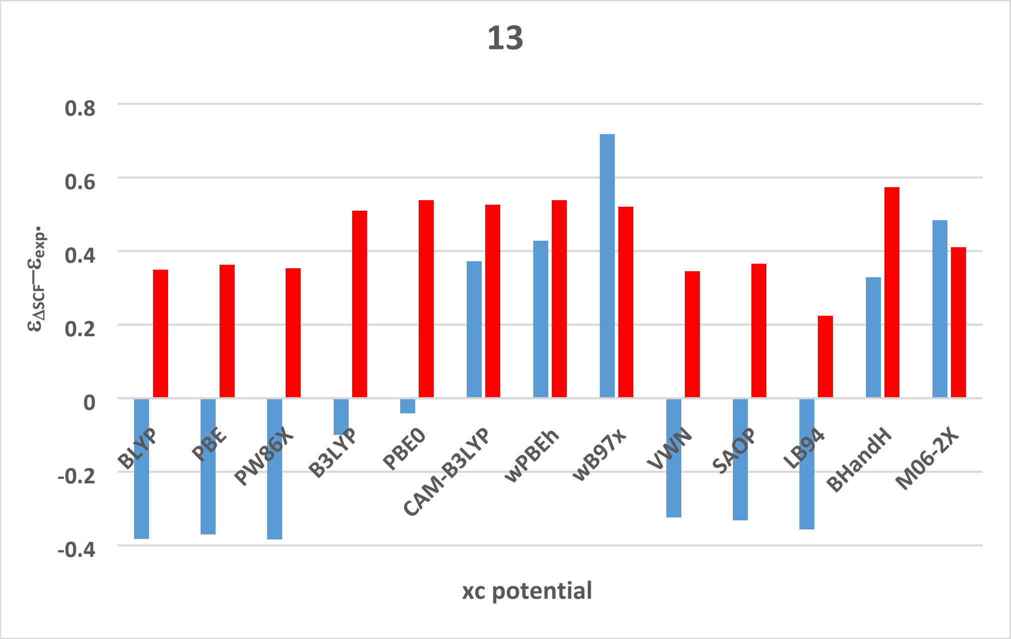

Figure S14. Box chart showing the comparison between ΔSCF (blue) and TDDFT (red) signed error of the first excitation energy with respect to the experimental value for the selection of DFT *xc* potentials for **13**.

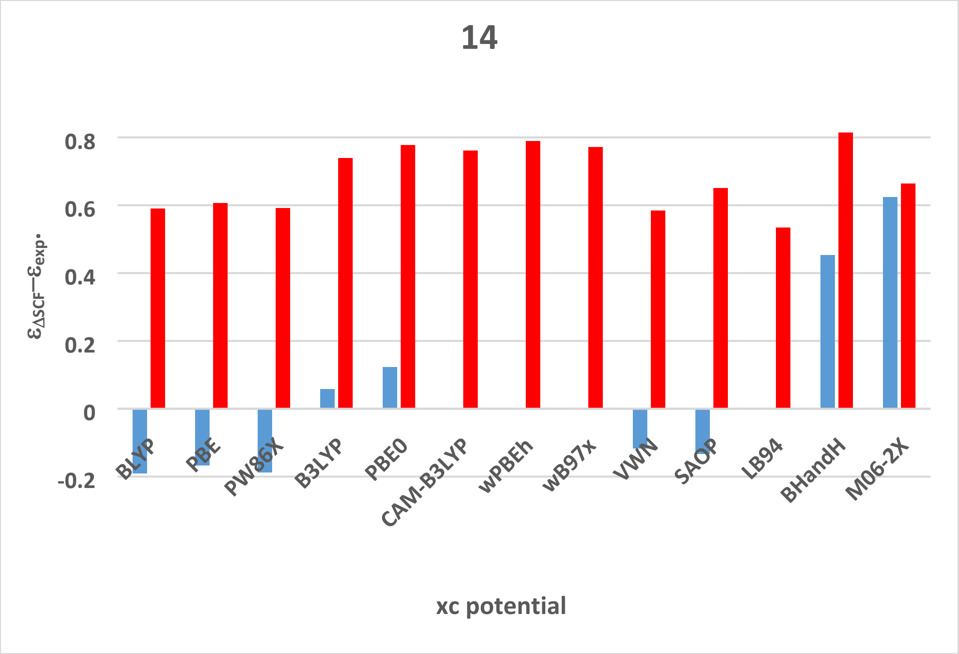

Figure S15. Box chart showing the comparison between ΔSCF (blue) and TDDFT (red) signed error of the first excitation energy with respect to the experimental value for the selection of DFT *xc* potentials for **14**.

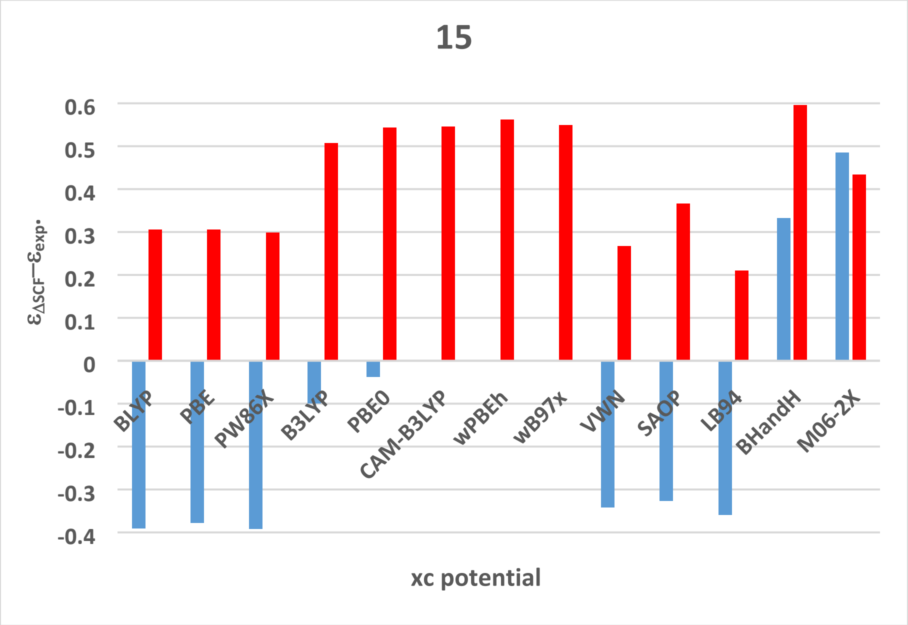

Figure S16. Box chart showing the comparison between ΔSCF (blue) and TDDFT (red) signed error of the first excitation energy with respect to the experimental value for the selection of DFT *xc* potentials for **15**.

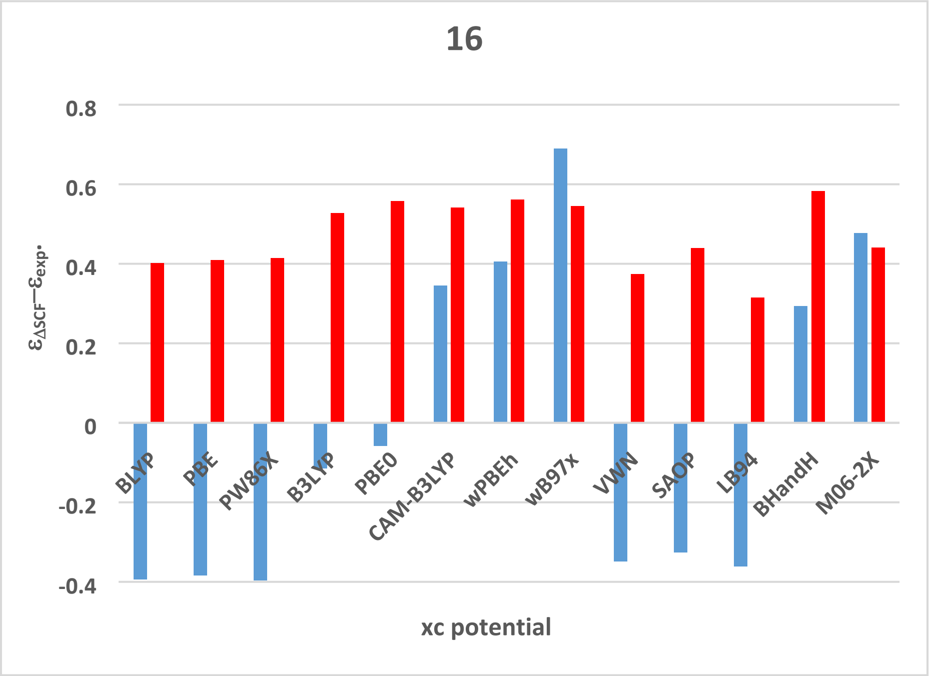

Figure S17. Box chart showing the comparison between ΔSCF (blue) and TDDFT (red) signed error of the first excitation energy with respect to the experimental value for the selection of DFT *xc* potentials for **16**.

Table S1. Comparison of the ωPBEh, ωB97x and CAM-B3LYP lowest TDDFT vertical energy transition for the entire molecular dataset, together with a statistical analysis of the results. Reported are the mean absolute error (MAE), maximum and minimum AE (Max AE and Min AE respectively), the standard deviation (SD) and the correlation coefficient (R^2) between theoretical vertical excitation energies and experimental values. Energies are in eV.

	**LRC-ωPBEh**	**LRC-ωB97x**	**CAM-B3LYP**
**1**	3.167	3.124	3.137
**2**	2.880	2.804	2.851
**3**	3.679	3.834	3.637
**4**	3.790	3.977	3.748
**5**	4.119	4.246	4.093
**5h**	4.488	4.609	4.467
**6**	3.827	3.898	3.799
**7**	3.185	3.210	3.178
**8**	3.492	3.599	3.475
**9**	2.970	2.959	2.963
**10**	3.576	3.629	3.545
**11**	2.660	2.634	2.648
**12**	3.451	3.548	3.428
**13**	2.950	2.932	2.938
**14**	3.143	3.124	3.115
**15**	2.984	2.971	2.968
**16**	2.879	2.862	2.859
**MAE**	0.559	0.579	0.541
**MAE[a]**	0.570	0.602	0.548
**max AE**	0.790	0.793	0.762
**max AE[a]**	0.790	0.793	0.762
**min AE**	0.350	0.198	0.329
**min AE[a]**	0.350	0.471	0.329
**SD**	0.121	0.136	0.116
**SD[a]**	0.117	0.101	0.116
**R^2**	0.862	0.889	0.858
**R^{2a}**	0.963	0.966	0.965

[a] Statistics obtained by removing molecule **4** from the dataset.

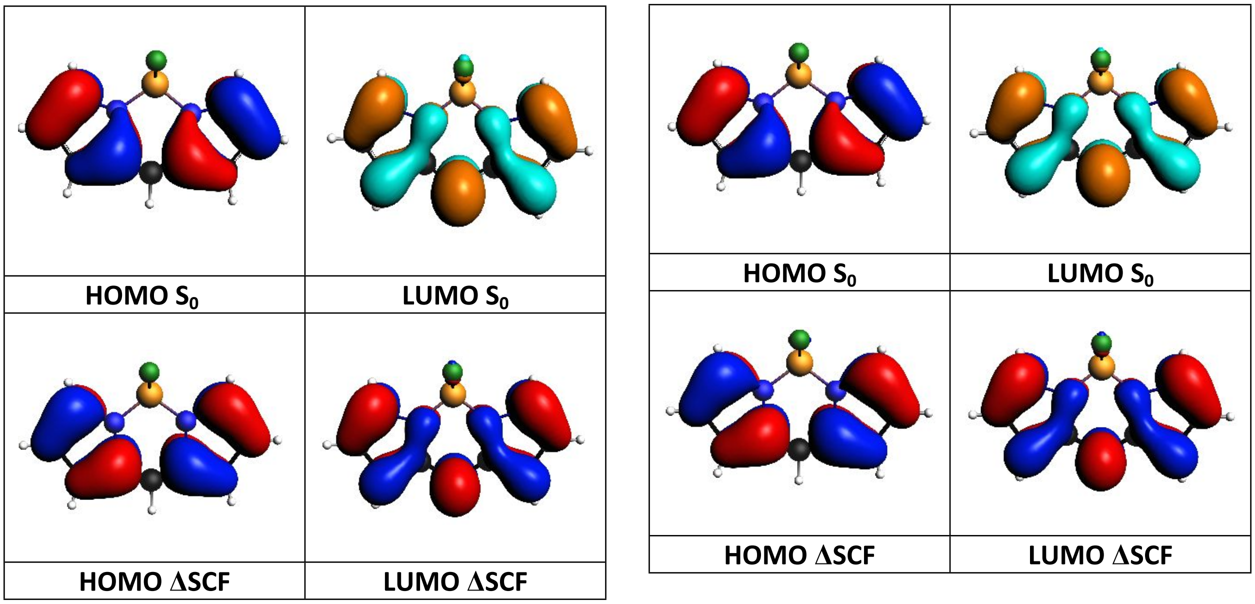

Figure S18. B3LYP (left panels) and PBE0 (right panels) HOMO and LUMO MO plots relative to a ground state KS run (upper panels) and to the corresponding ΔSCF run (lower panels) for **1**. Isosurfaces are drawn with a isovalue of 0.03 e/$Å^{3/2}$.

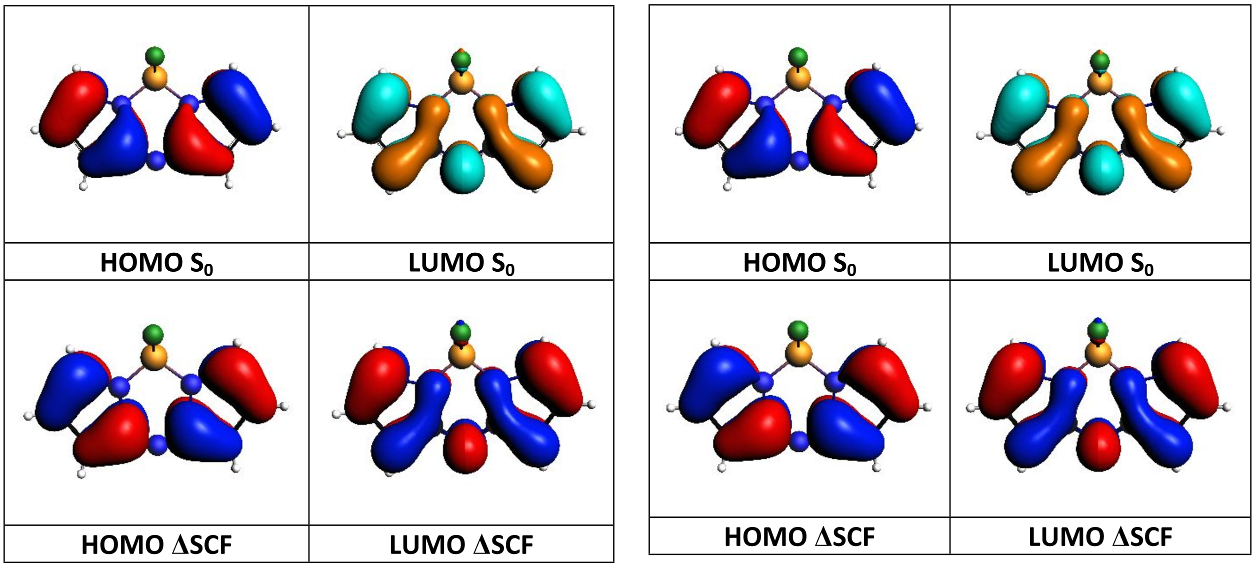

Figure S19. B3LYP (left panels) and PBE0 (right panels) HOMO and LUMO MO plots relative to a ground state KS run (upper panels) and to the corresponding ΔSCF run (lower panels) for **2**. Isosurfaces are drawn with a isovalue of 0.03 e/$Å^{3/2}$.

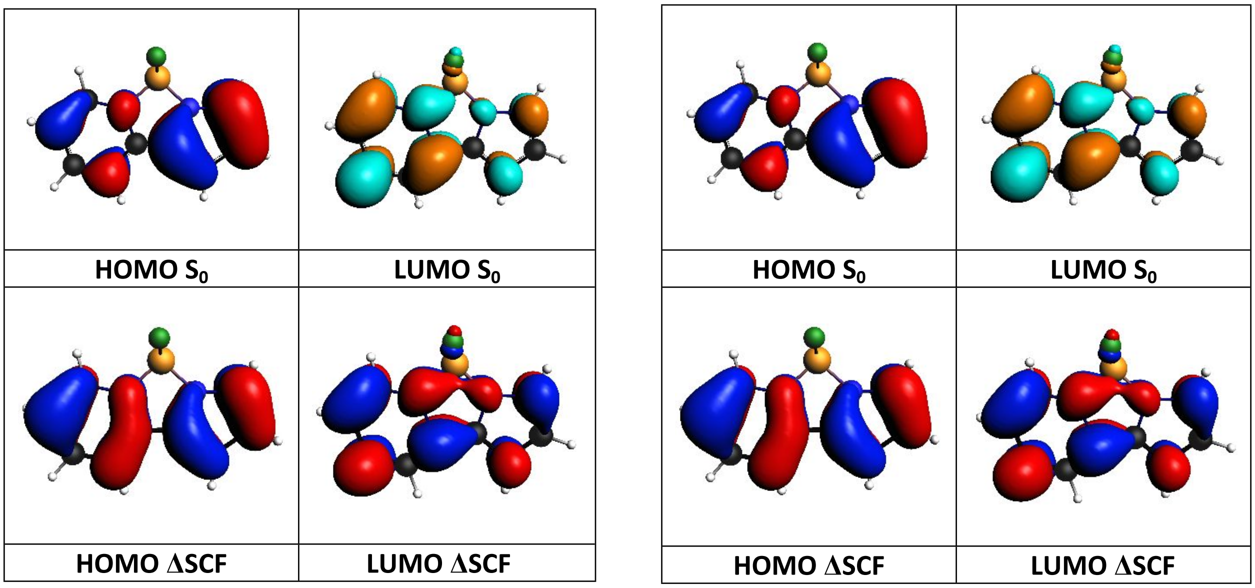

Figure S20. B3LYP (left panels) and PBE0 (right panels) HOMO and LUMO MO plots relative to a ground state KS run (upper panels) and to the corresponding ΔSCF run (lower panels) for **3**. Isosurfaces are drawn with a isovalue of 0.03 e/$Å^{3/2}$.

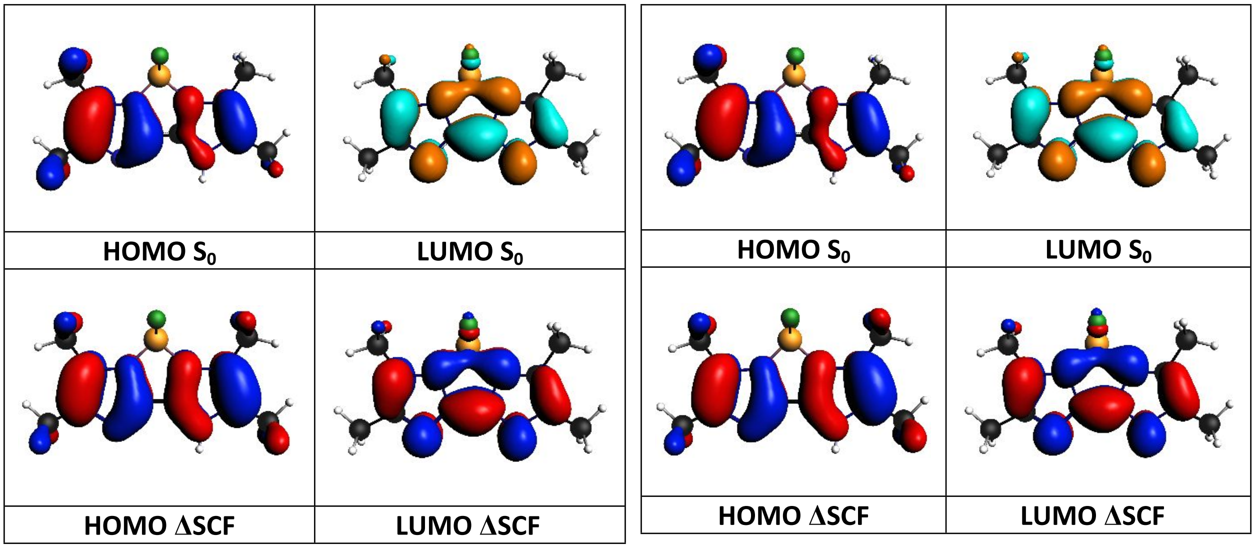

Figure S21. B3LYP (left panels) and PBE0 (right panels) HOMO and LUMO MO plots relative to a ground state KS run (upper panels) and to the corresponding ΔSCF run (lower panels) for **5**. Isosurfaces are drawn with a isovalue of 0.03 e/Å$^{3/2}$.

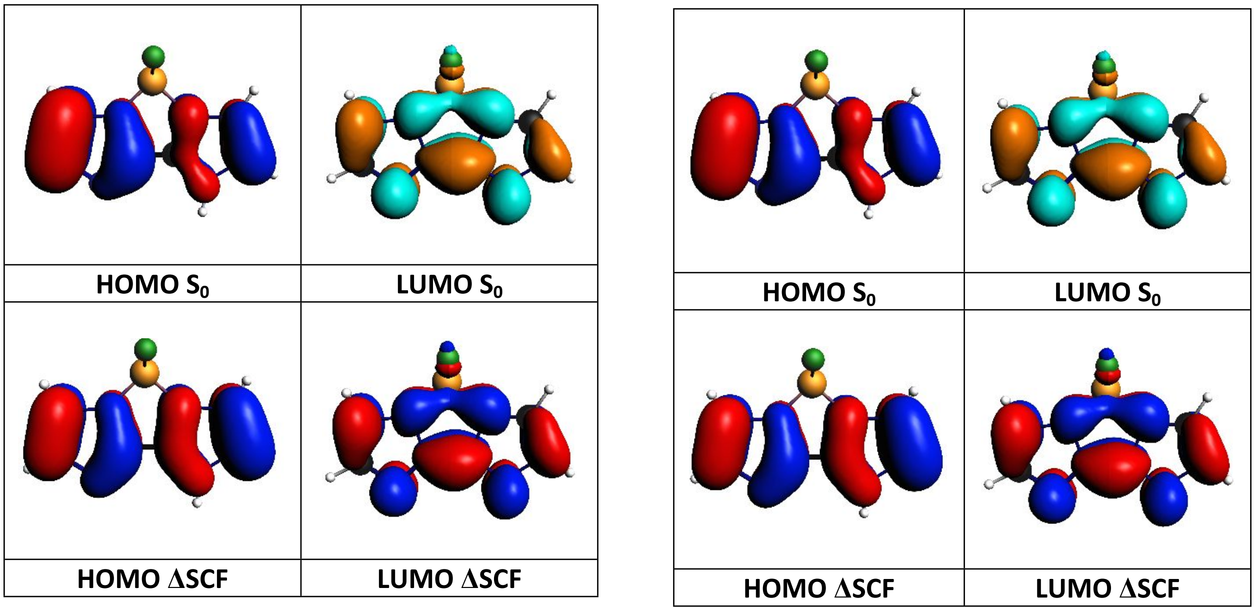

Figure S22. B3LYP (left panels) and PBE0 (right panels) HOMO and LUMO MO plots relative to a ground state KS run (upper panels) and to the corresponding ΔSCF run (lower panels) for **5h**. Isosurfaces are drawn with a isovalue of 0.03 e/Å$^{3/2}$.

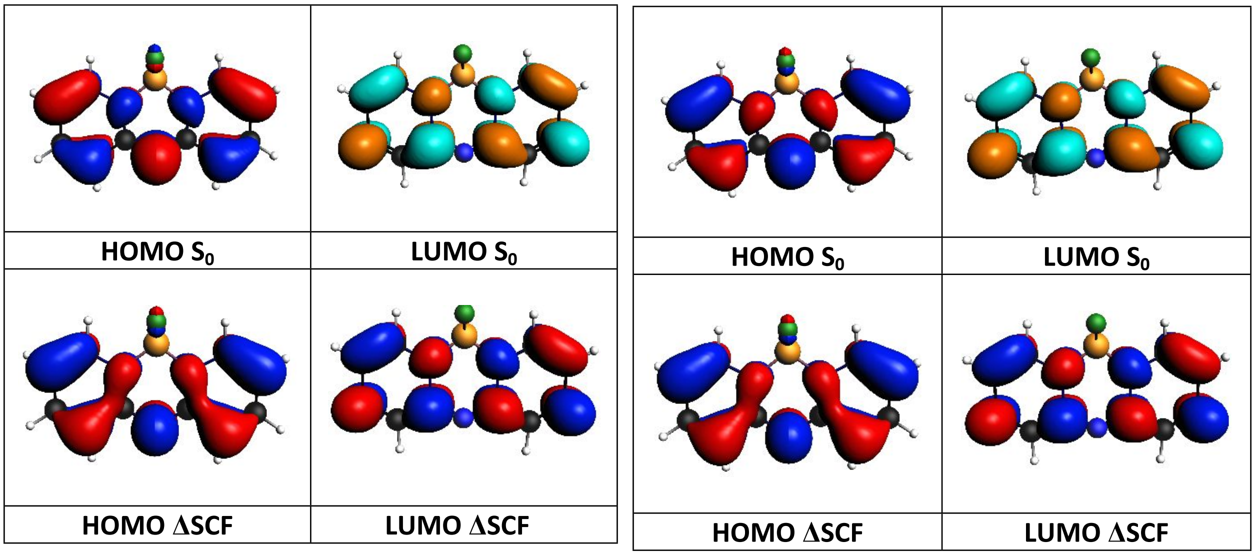

Figure S23. B3LYP (left panels) and PBE0 (right panels) HOMO and LUMO MO plots relative to a ground state KS run (upper panels) and to the corresponding ΔSCF run (lower panels) for **6**. Isosurfaces are drawn with a isovalue of 0.03 e/$Å^{3/2}$.

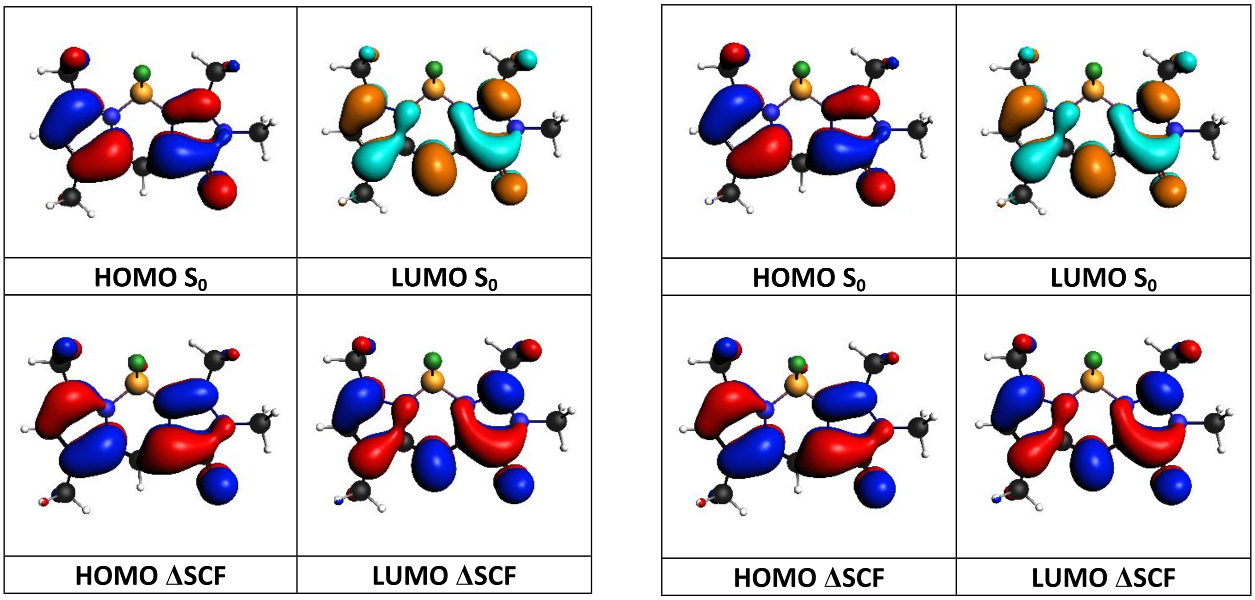

Figure S24. B3LYP (left panels) and PBE0 (right panels) HOMO and LUMO MO plots relative to a ground state KS run (upper panels) and to the corresponding ΔSCF run (lower panels) for **7**. Isosurfaces are drawn with a isovalue of 0.03 e/$Å^{3/2}$.

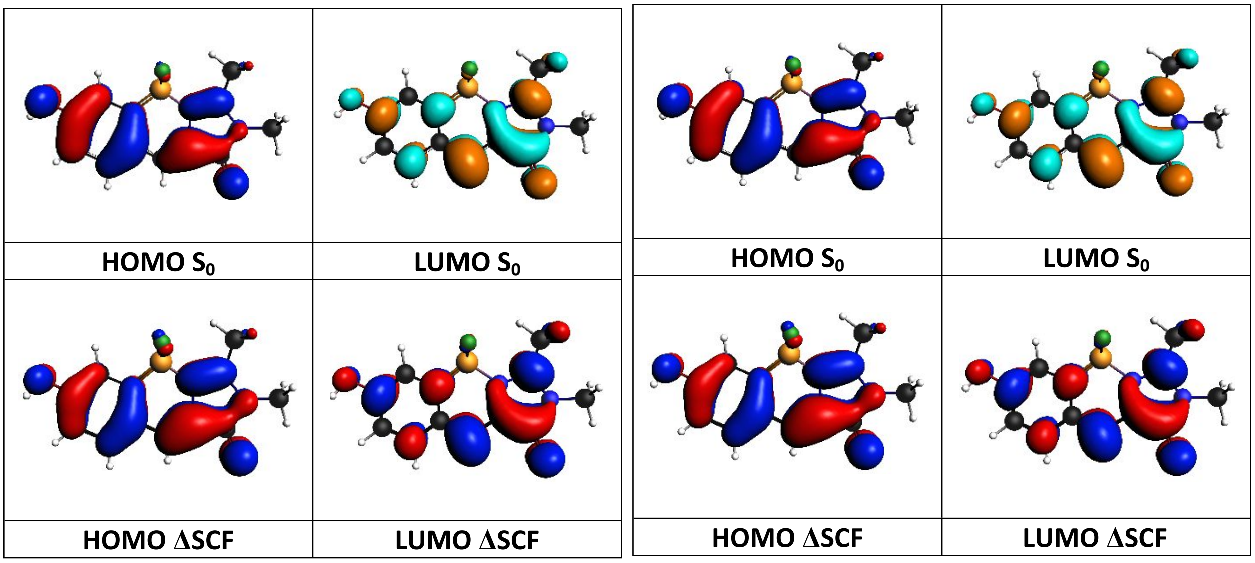

Figure S25. B3LYP (left panels) and PBE0 (right panels) HOMO and LUMO MO plots relative to a ground state KS run (upper panels) and to the corresponding ΔSCF run (lower panels) for **8**. Isosurfaces are drawn with a isovalue of 0.03 e/$Å^{3/2}$.

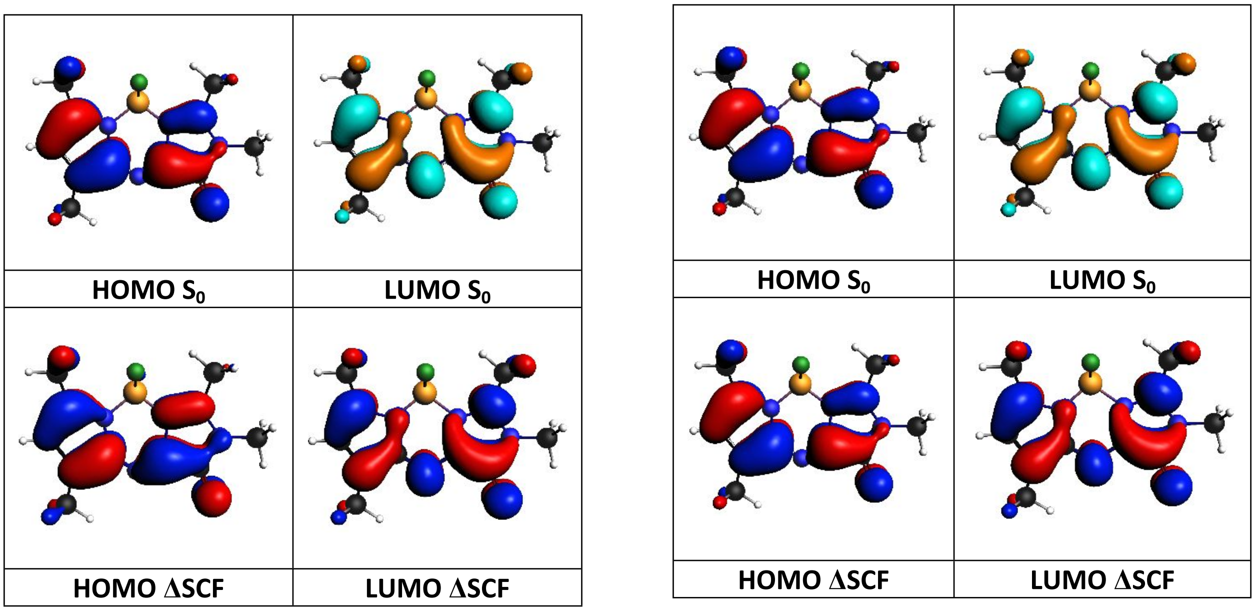

Figure S26. B3LYP (left panels) and PBE0 (right panels) HOMO and LUMO MO plots relative to a ground state KS run (upper panels) and to the corresponding ΔSCF run (lower panels) for **9**. Isosurfaces are drawn with a isovalue of 0.03 e/$Å^{3/2}$.

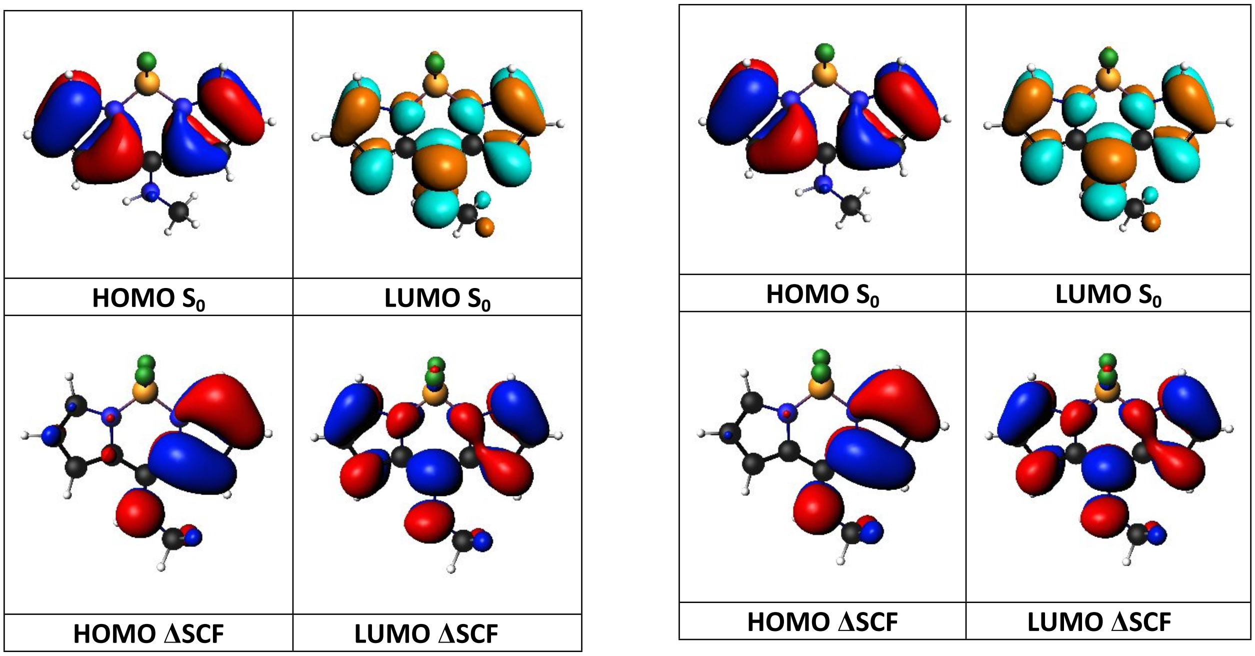

Figure S27. B3LYP (left panels) and PBE0 (right panels) HOMO and LUMO MO plots relative to a ground state KS run (upper panels) and to the corresponding ΔSCF run (lower panels) for **10**. Isosurfaces are drawn with a isovalue of 0.03 e/$Å^{3/2}$.

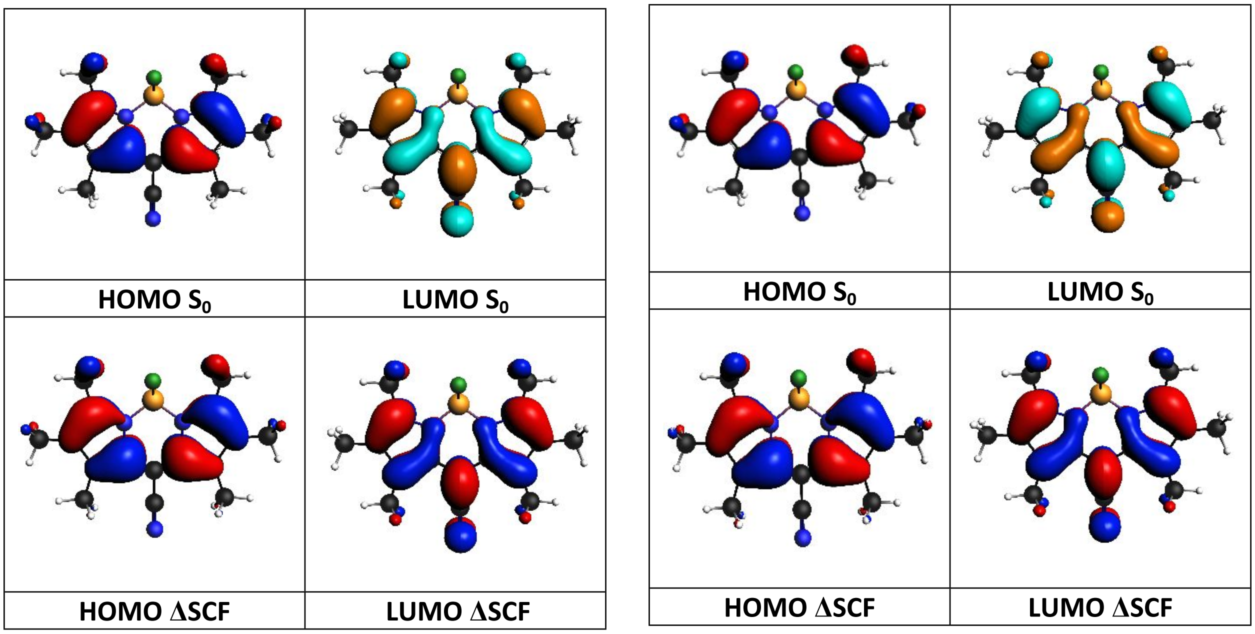

Figure S28. B3LYP (left panels) and PBE0 (right panels) HOMO and LUMO MO plots relative to a ground state KS run (upper panels) and to the corresponding ΔSCF run (lower panels) for **11**. Isosurfaces are drawn with a isovalue of 0.03 e/Å$^{3/2}$.

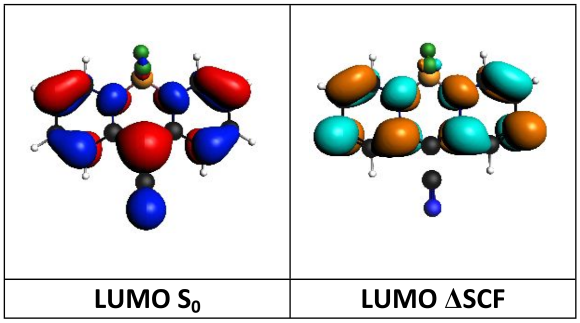

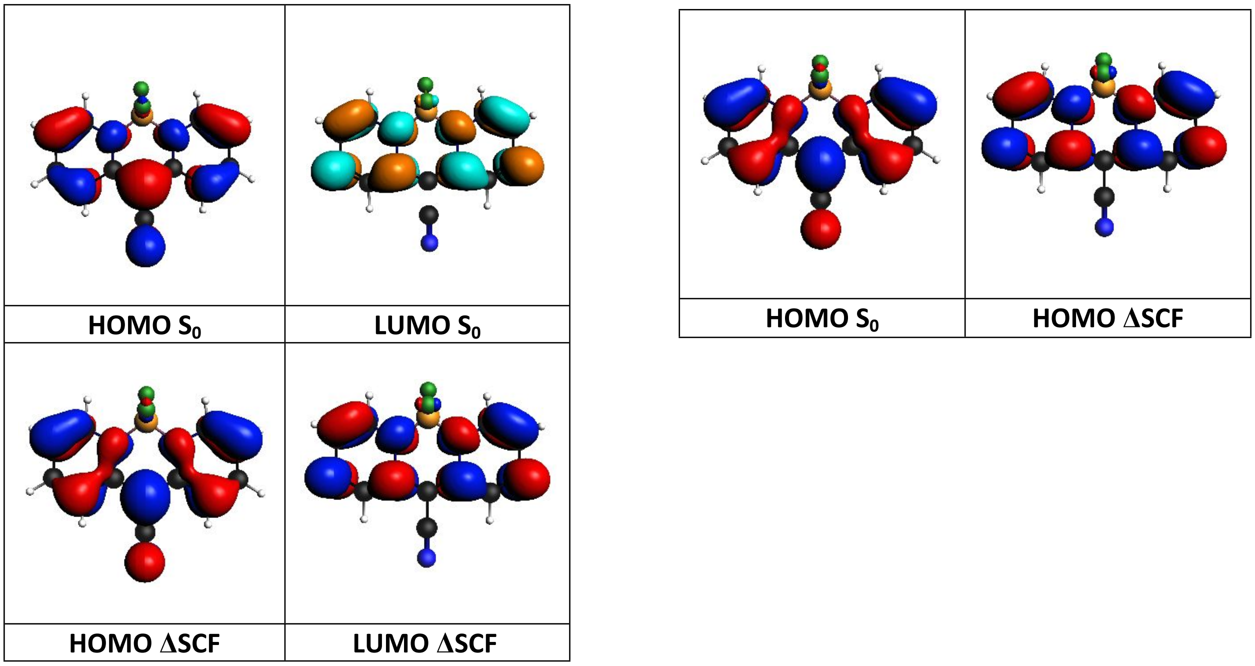

Figure S29. B3LYP (left panels) and PBE0 (right panels) HOMO and LUMO MO plots relative to a ground state KS run (upper panels) and to the corresponding ΔSCF run (lower panels) for **12**. Isosurfaces are drawn with a isovalue of 0.03 e/Å$^{3/2}$.

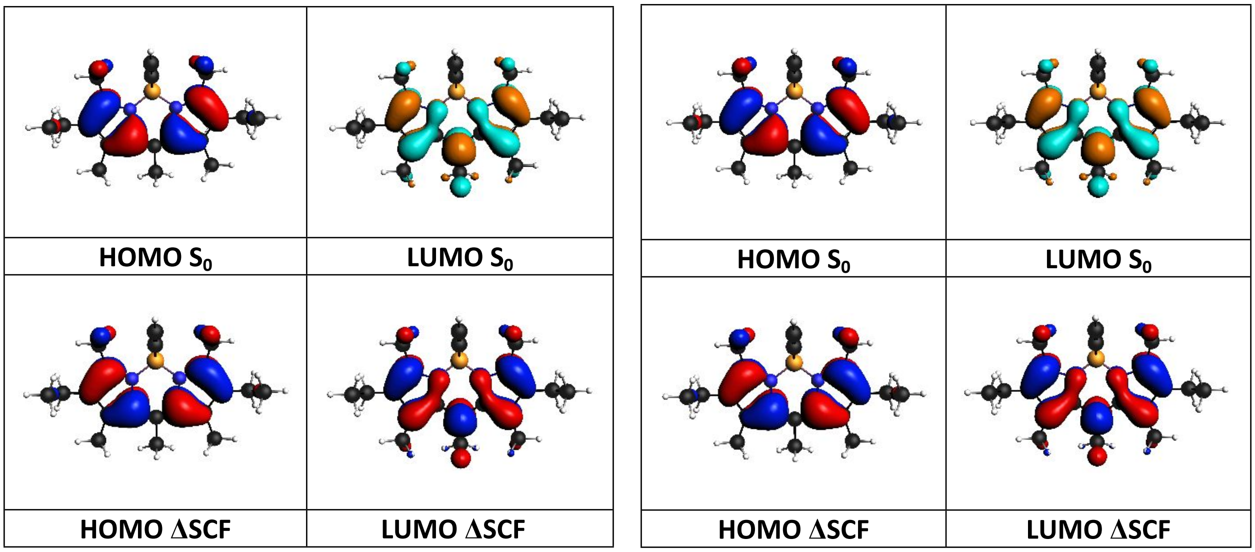

Figure S30. B3LYP (left panels) and PBE0 (right panels) HOMO and LUMO MO plots relative to a ground state KS run (upper panels) and to the corresponding ΔSCF run (lower panels) for **13**. Isosurfaces are drawn with a isovalue of 0.03 e/Å$^{3/2}$.

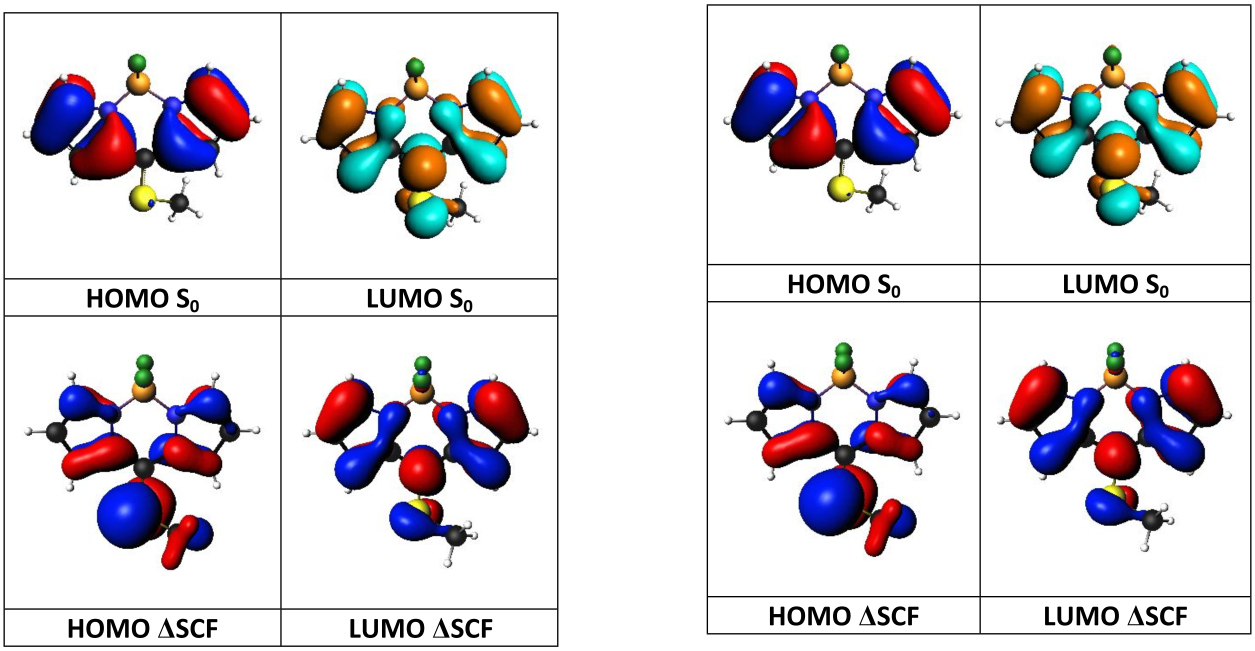

Figure S31. B3LYP (left panels) and PBE0 (right panels) HOMO and LUMO MO plots relative to a ground state KS run (upper panels) and to the corresponding ΔSCF run (lower panels) for **14**. Isosurfaces are drawn with a isovalue of 0.03 e/$Å^{3/2}$.

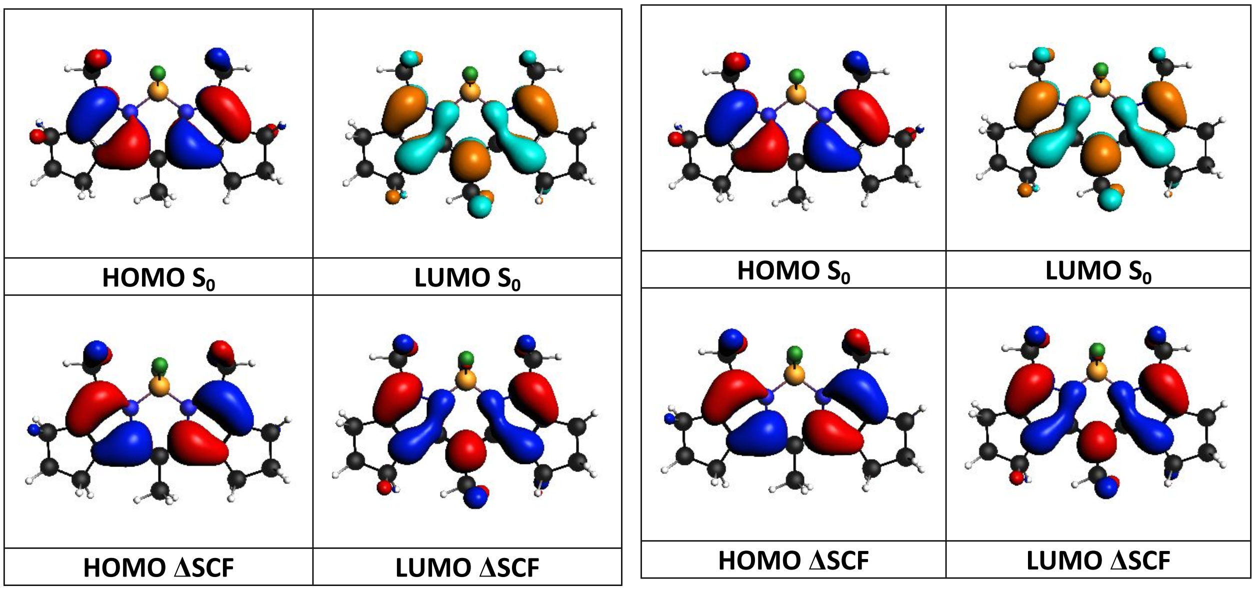

Figure S32. B3LYP (left panels) and PBE0 (right panels) HOMO and LUMO MO plots relative to a ground state KS run (upper panels) and to the corresponding ΔSCF run (lower panels) for **15**. Isosurfaces are drawn with a isovalue of 0.03 e/$Å^{3/2}$.

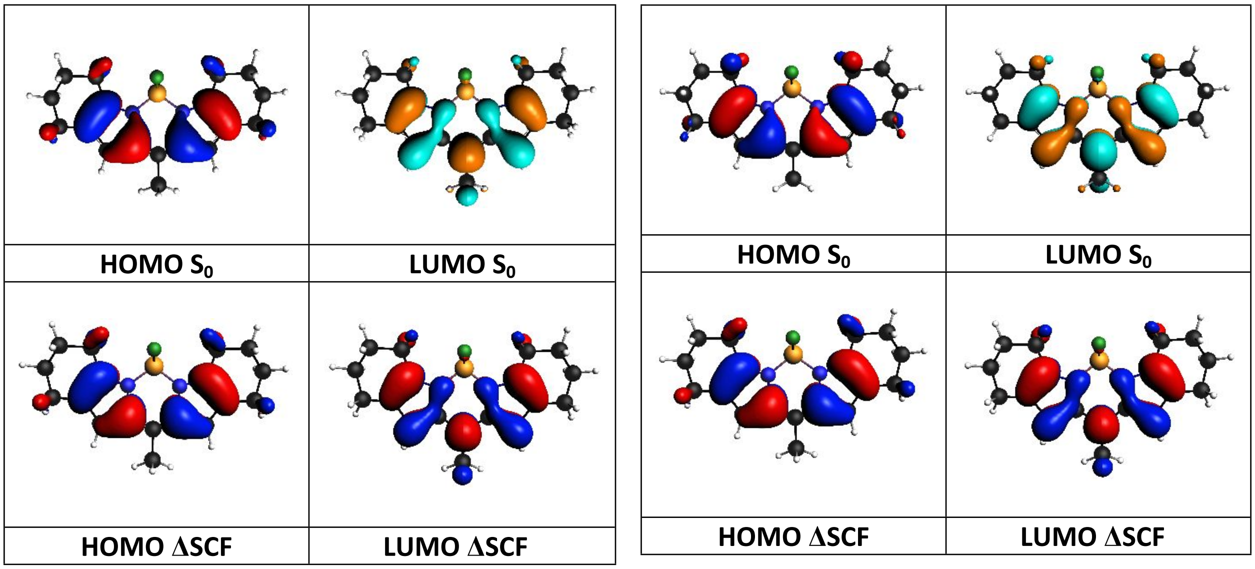

Figure S33. B3LYP (left panels) and PBE0 (right panels) HOMO and LUMO MO plots relative to a ground state KS run (upper panels) and to the corresponding ΔSCF run (lower panels) for **16**. Isosurfaces are drawn with a isovalue of 0.03 e/Å$^{3/2}$.

Table S2. ΔSCF and TDDFT vertical excitation energies and oscillator strength for the HOMO→LUMO transition of **2**, and for a selection of DFT *xc* potentials. The differences with respect to the experimental value of 2.252 eV is also reported in parenthesis. The CASPT2/cc-pVDZ value of the vertical excitation energy is 2.344 eV [1]. For TDDFT the eigenvector corresponding to the lowest energy root is also reported (H: HOMO, L:LUMO).

	**ΔSCF**		**TDDFT**		
**XC**	**ε(eV)**	**f**	**ε(eV)**	**f**	**dominant excitations**
**BLYP**	1.885 (-0.367)	0.48	2.7283 (0.476)	0.098	0.3939 H→L + 0.5947 (H-1) →L
**PBE**	1.912 (-0.34)	0.48	2.7397 (0.488)	0.0094	0.3852 H→L + 0.6035 (H-1) →L
**PW86X**	1.901 (-0.351)	0.48	2.7253 (0.473)	0.094	0.3882 H→L + 0.6005 (H-1) →L
**B3LYP**	2.200 (-0.0518)	0.53	2.8884 (0.636)	0.31	0.7416 H→L + 0.2461 (H-1) →L
**PBE0**	2.288 (0.0361)	0.53	2.9195 (0.668)	0.35	0.7964 H→L + 0.1904 (H-1) →L
**CAM-B3LYP**	2.711 (0.459)	0.58	2.851 (0.599)	0.47	0.9125 H→L + 0.0670 (H-1) →L
**wPBEh**	2.776 (0.524)	0.55	2.881 (0.628)	0.46	0.9069 H→L + 0.0710 (H-1) →L
**wB97x**	3.070 (0.818)	0.61	2.804 (0.552)	0.51	0.9367 H→L + 0.0378 (H-1) →L
**VWN**	1.921 (-0.331)	0.49	2.7102 (0.458)	0.088	0.3832 H→L + 0.6056 (H-1) →L

**SAOP**	1.957 (-0.295)	0.51	2.7575 (0.506)	0.14	0.4866 H→L + 0.5021 (H-1) →L
**LB94**	1.927 (-0.326)	0.50	2.6427 (0.391)	0.010	0.4371 H→L + 0.5510 (H-1) →L
**BHandH**	2.639 (0.387)	0.58	2.8935 (0.642)	0.50	0.9263 H→L + 0.0557 (H-1) →L
**M06-2X**	2.801 (0.549)	0.63	2.7652 (0.642)	0.46	0.9227 H→L + 0.0557 (H-1) →L

Table S3. ΔSCF and TDDFT vertical excitation energies and oscillator strength for the HOMO→LUMO transition of **3**, and for a selection of DFT *xc* potentials. The differences with respect to the experimental value of 3.259 eV is also reported in parenthesis. The CASPT2/cc-pVDZ value of the vertical excitation energy is 3.129 eV [1]. For TDDFT the eigenvector corresponding to the lowest energy root is also reported (H: HOMO, L:LUMO).

	**ΔSCF**		**TDDFT**		
**XC**	**ε(eV)**	**f**	**ε(eV)**	**f**	**dominant excitations**
**BLYP**	2.892 (-0.368)	0.32	2.796 (-0.463)	0.038	0.8328 H→L + 0.1370 H → (L+1)
**PBE**	2.915 (-0.344)	0.32	2.816 (-0.443)	0.037	0.8337 H→L + 0.1368 H → (L+1)
**PW86X**	2.893 (-0.366)	0.32	2.803 (-0.456)	0.037	0.8339 H→L + 0.1361 H → (L+1)
**B3LYP**	3.135 (-0.124)	0.33	3.216 (-0.0433)	0.099	0.9005 H→L + 0.0844 H → (L+1)
**PBE0**	3.196 (-0.0629)	0.33	3.330 (0.0705)	0.12	0.9179 H→L + 0.0675 H → (L+1)
**CAM-B3LYP**	3.479 (0.220)	0.33	3.637 (0.378)	0.25	0.9655 H→L+ 0.0036 H → (L+1)
**wPBEh**	-	-	3.679 (0.420)	0.25	0.9628 H→L
**wB97x**	-	-	3.834 (0.575)	0.32	0.9455 H→L + 0.0162 (H-2) →L
**VWN**	2.943 (-0.316)	0.33	2.789 (-0.470)	0.036	0.8363 H→L + 0.1342 H → (L+1)
**SAOP**	2.972 (-0.287)	0.33	2.864 (-0.395)	0.047	0.8337 H→L + 0.1372 H → (L+1)
**LB94**	2.935 (-0.324)	0.34	2.659 (-0.601)	0.036	0.8364 H→L + 0.1326 H → (L+1)
**BHandH**	3.487 (0.228)	0.35	3.672 (0.413)	0.25	0.9691 H→L + 0.0055 H → (L+1)
**M06-2X**	3.738 (0.479)	0.35	3.5621 (0.433)	0.24	0.9666 H→ L + 0.0075 (H-2) → L

Table S4. ΔSCF and TDDFT vertical excitation energies and oscillator strength for the HOMO→LUMO transition of **4**, and for a selection of DFT *xc* potentials. The differences with respect to the experimental value of 4.175 eV is also reported in parenthesis. The CASPT2/cc-pVDZ value of the vertical excitation energy is 3.278 eV [1]. For TDDFT the eigenvector corresponding to the lowest energy root is also reported (H: HOMO, L:LUMO).

	**ΔSCF**		**TDDFT**		
**XC**	**ε(eV)**	**f**	**ε(eV)**	**f**	**dominant excitations**
**BLYP**	3.023 (-1.152)	0.29	2.835 (-1.34)	0.045	0.8442 H → L + 0.1374 H → (L+1)
**PBE**	3.042 (-1.133)	0.29	2.851 (-1.324)	0.044	0.8449 H→ L + 0.1369 H → (L+1)
**PW86X**	3.023 (-1.152)	0.29	2.841 (-1.334)	0.044	0.8450 H→ L + 0.1367 H → (L+1)
**B3LYP**	3.265 (-0.910)	0.31	3.277 (-0.898)	0.11	0.9240 H→ L + 0.0644 H → (L+1)
**PBE0**	3.319 (-0.857)	0.30	3.393 (-0.782)	0.13	0.9407 H→ L + 0.0477 H → (L+1)
**CAM-B3LYP**	3.592 (-0.583)	0.314	3.748 (-0.427)	0.25	0.9671 H→ L
**wPBEh**	-	-	3.790 (-0.385)	0.25	0.9637 H→ L
**wB97x**	-	-	3.977 (-0.198)	0.31	0.9455 H→ L + 0.0162 (H-2) → L
**VWN**	3.073 (-1.102)	0.30	2.835 (-1.340)	0.043	0.8467 H→ L + 0.1352 H → (L+1)
**SAOP**	3.113 (-1.062)	0.30	2.891 (-1.284)	0.054	0.8419 H→ L + 0.1397 H → (L+1)
**LB94**	3.066 (-1.109)	0.31	2.712 (-1.464)	0.044	0.8432 H→ L + 0.1387 H → (L+1)
**BHandH**	3.591 (-0.584)	0.32	3.772 (-0.403)	0.25	0.9723 H→ L + 0.0004 H → (L+1)
**M06-2X**	3.628 (-0.547)	0.37	3.675 (-0.397)	0.24	0.9653 H→L + 0.0095 (H-3) → L

Table S5. ΔSCF and TDDFT vertical excitation energies and oscillator strength for the HOMO→LUMO transition of **5**, and for a selection of DFT *xc* potentials. The differences with respect to the experimental value of 3.712 eV is also reported in parenthesis. The CASPT2/cc-pVDZ value of the vertical excitation energy is 3.726 eV [1]. For TDDFT the eigenvector corresponding to the lowest energy root is also reported (H: HOMO, L:LUMO).

	**ΔSCF**		**TDDFT**		
**XC**	**ε(eV)**	**f**	**ε(eV)**	**f**	**dominant excitations**
**BLYP**	3.197 (0.515)	0.38	3.654 (-0.0577)	0.45	0.9727 H→L + 0.0043 (H-1) $^-$→L
**PBE**	3.222 (0.490)	0.39	3.675 (-0.0371)	0.44	0.9729 H→L + 0.0043 (H-1) $^-$→L
**PW86X**	3.201 (0.512)	0.38	3.659 (-0.0534)	0.44	0.9730 H→L + 0.0044 (H-1) $^-$→L
**B3LYP**	3.525 (0.187)	0.40	3.876 (0.164)	0.48	0.9802 H→L + 0.027 (H-2) $^-$→L
**PBE0**	3.623 (0.0888)	0.40	3.952 (0.240)	0.49	0.9802 H→L + 0.0029 (H-2) $^-$→L
**CAM-B3LYP**	3.974 (0.262)	0.41	4.093 (0.381)	0.49	0.9716 H→L + 0.041 H$^-$→ (L+1)
**wPBEh**	-	-	4.119 (0.407)	0.48	0.9700 H→L
**wB97x**	-	-	4.246 (0.534)	0.48	0.9606 H→L
**VWN**	3.209 (0.503)	0.39	3.627 (-0.0853)	0.44	0.9730 H→L + 0.0028 H $^-$→ (L+1)
**SAOP**	3.339 (0.373)	0.40	3.664 (-0.0483)	0.46	0.9758 H→L + 0.0044 (H-2) $^-$→ L
**LB94**	3.237 (0.475)	0.40	3.484 (-0.2280)	0.43	0.9740 H→L + 0.0046 (H-2) $^-$→ L
**BHandH**	3.989 (0.277)	0.43	4.129 (0.417)	0.51	0.9740 H→L + 0.0032 H $^-$→ (L+1)
**M06-2X**	4.097 (0.385)	0.44	4.043 (0.331)	0.48	0.9685 H→L

Table S6. ΔSCF and TDDFT vertical excitation energies and oscillator strength for the HOMO→LUMO transition of **5h**, and for a selection of DFT *xc* potentials. The differences with respect to the experimental value of 4.138 eV is also reported in parenthesis. The CASPT2/cc-pVDZ value of the vertical excitation energy is 4.104 eV. [1]. For TDDFT the eigenvector corresponding to the lowest energy root is also reported (H: HOMO, L:LUMO).

	**ΔSCF**		**TDDFT**		
**XC**	**ε(eV)**	**f**	**ε(eV)**	**f**	**dominant excitations**
**BLYP**	3.555 (-0.583)	0.38	4.059 (-0.0791)	0.36	0.9648 H→L + 0.0082 (H-1) → L
**PBE**	3.588 (-0.550)	0.38	4.088 (-0.0499)	0.35	0.9644 H→L + 0.0082 (H-1) → L
**PW86X**	3.566 (-0.572)	0.38	4.071 (-0.0674)	0.36	0.9647 H→L + 0.0083 (H-1) → L
**B3LYP**	3.891 (-0.247)	0.39	4.267 (0.129)	0.39	0.9737 H→L + 0.0046 (H-1) → L
**PBE0**	3.996 (-0.142)	0.40	4.344 (0.206)	0.40	0.9742 H→L + 0.0044 (H-1) →L
**CAM-B3LYP**	4.338 (0.200)	0.41	4.467 (0.329)	0.40	0.9687 H→L + 0.0054 H→ (L+1)
**wPBEh**	-	-	4.488 (0.350)	0.40	0.9679 H→L
**wB97x**	-	-	4.609 (0.471)	0.40	0.9608 H→L
**VWN**	3.596 (-0.542)	0.39	4.063 (-0.0751)	0.35	0.9636 H→L + 0.0082 H-1) → L
**SAOP**	3.685 (-0.453)	0.39	4.075 (-0.0633)	0.37	0.9691 H→L + 0.0061 H-1) → L
**LB94**	3.601 (-0.540)	0.40	3.903 (-0.235)	0.34	0.9654 H→L + 0.0081 H-1) → L
**BHandH**	4.387 (0.250)	0.43	4.524 (0.386)	0.42	0.9700 H→L + 0.0047 H → (L+1)
**M06-2X**	4.660 (0.522)	0.44	4.412 (0.274)	0.38	0.9700 H→L + 0.0047 H → (L+1)

Table S7. ΔSCF and TDDFT vertical excitation energies and oscillator strength for the HOMO→LUMO transition of **6**, and for a selection of DFT *xc* potentials. The differences with respect to the experimental value of 3.125 eV is also reported in parenthesis. The CASPT2/cc-pVDZ value of the vertical excitation energy is 2.821 eV. [1]. For TDDFT the eigenvector corresponding to the lowest energy root is also reported (H: HOMO, L:LUMO).

	**ΔSCF**		**TDDFT**		
**XC**	**ε(eV)**	**f**	**ε(eV)**	**f**	**dominant excitations**
**BLYP**	2.755 (-0.370)	0.36	3.290 (0.165)	0.19	0.8741 H→ L + 0.0997 H→ (L+1)
**PBE**	2.773 (-0.352)	0.36	3.310 (0.185)	0.19	0.8737 H→L + 0.1003 H → (L+1)
**PW86X**	2.748 (-0.377)	0.36	3.293 (0.168)	0.19	0.8731 H→L + 0.1009 H→ (L+1)
**B3LYP**	3.048 (-0.0774)	0.39	3.595 (0.470)	0.32	0.9595 H→L + 0.0253 H→ (L+1)
**PBE0**	3.101 (-0.0238)	0.39	3.677 (0.552)	0.35	0.9654 H→L + 0.0172 H→ (L+1)
**CAM-B3LYP**	3.472 (0.347)	0.39	3.799 (0.674)	0.42	0.9551 H→L + 0.0017 H→ (L+1)
**wPBEh**	3.525 (0.400)	0.38	3.827 (0.702)	0.42	0.9505 H→L + 0.0201 (H-2)→ L
**wB97x**	3.795 (0.670)	0.39	3.898 (0.773)	0.45	0.9346 H→L + 0.0306 (H-1)→ (L+1)
**VWN**	2.824 (-0.301)	0.37	3.286 (0.161)	0.19	0.8710 H→L + 0.1031 H→ (L+1)
**SAOP**	2.848 (-0.278)	0.36	3.382 (0.257)	0.21	0.8949 H→L + 0.0769 H→ (L+1)
**LB94**	2.803 (-0.322)	0.38	3.237 (0.112)	0.20	0.8715 H→L + 0.1003 H→ (L+1)
**BHandH**	3.444 (0.319)	0.40	3.880 (0.755)	0.44	0.9594 H→L + 0.0010 H→ (L+1)
**M06-2X**	3.690 (0.565)	0.45	3.723 (0.598)	0.42	0.9543 H→L + 0.0182 (H-1)→ (L+1)

Table S8. ΔSCF and TDDFT vertical excitation energies and oscillator strength for the HOMO→LUMO transition of **7**, and for a selection of DFT *xc* potentials. The differences with respect to the experimental value of 2.583 eV is also reported in parenthesis [1]. For TDDFT the eigenvector corresponding to the lowest energy root is also reported (H: HOMO, L:LUMO).

	**ΔSCF**		**TDDFT**		
**XC**	**ε(eV)**	**f**	**ε(eV)**	**f**	**dominant excitations**
**BLYP**	2.097 (-0.486)	0.52	2.994 (0.411)	0.51	0.9087 H→ L + 0.0457 (H-1) → L
**PBE**	2.108 (-0.476)	0.52	3.008 (0.425)	0.52	0.9165 H→ L + 0.0391 (H-1) → L
**PW86X**	2.098 (-0.485)	0.52	2.997 (0.414)	0.51	0.9125 H→ L + 0.0428 (H-1) → L
**B3LYP**	2.485 (-0.0985)	0.56	3.127 (0.544)	0.62	0.9666 H→ L + 0.0109 (H-1) → L
**PBE0**	2.576 (-0.0072)	0.57	3.165 (0.528)	0.64	0.9724 H→ L + 0.080 (H-1) → L
**CAM-B3LYP**	-	-	3.178 (0.595)	0.68	0.9763 H→ L + 0.0040 (H-1) → L
**wPBEh**	-	-	3.185 (0.602)	0.68	0.9759 H→ L
**wB97x**	-	-	3.210 (0.627)	0.70	0.9708 H→ L
**VWN**	2.115 (-0.469)	0.53	2.987 (0.404)	0.52	0.9197 H→ L + 0.0363 (H-1) → L
**SAOP**	2.176 (-0.407)	0.53	3.055 (0.472)	0.54	0.9212 H→ L + 0.0341 (H-1) → L
**LB94**	2.123 (-0.460)	0.54	2.914 (0.331)	0.48	0.8845 H→ L + 0.0703 (H-1) → L
**BHandH**	3.051 (0.468)	0.62	3.241 (0.658)	0.71	0.9794 H→ L + 0.0030 (H-1) → L
**M06-2X**	3.120 (0.537)	0.65	3.075 (0.492)	0.67	0.9777 H→L + 0.0033(H-1) → L

Table S9. ΔSCF and TDDFT vertical excitation energies and oscillator strength for the HOMO→LUMO transition of **8**, and for a selection of DFT *xc* potentials. The differences with respect to the experimental value of 2.995 eV is also reported in parenthesis. . The CASPT2/cc-pVDZ value of the vertical excitation energy is 3.142 eV [1]. For TDDFT the eigenvector corresponding to the lowest energy root is also reported (H: HOMO, L:LUMO).

	**ΔSCF**		**TDDFT**		
**XC**	**ε(eV)**	**f**	**ε(eV)**	**f**	**dominant excitations**
**BLYP**	2.329 (-0.666)	0.54	3.021 (0.0262)	0.54	0.9351 H→L + 0.0171 (H-2) → L
**PBE**	2.338 (-0.657)	0.54	3.032 (0.037)	0.54	0.9374 H→L + 0.0153 (H-2) → L
**PW86X**	2.329 (-0.666)	0.54	3.021 (0.026)	0.54	0.9367 H→L + 0.0157(H-2) → L
**B3LYP**	2.807 (-0.188)	0.57	3.267 (0.272)	0.63	0.9735 H→L + 0.0065 H → (L+1)
**PBE0**	2.932 (-0.0635)	0.58	3.333 (0.338)	0.65	0.9772 H→L + 0.0051 H → (L+1)
**CAM-B3LYP**	3.413 (0.418)	0.60	3.475 (0.480)	0.70	0.9697 H→L + 0.0057 (H-1) → L
**wPBEh**	-	-	3.492 (0.497)	0.69	0.9637 H→L
**wB97x**	-	-	3.599 (0.604)	0.72	0.9504 H→L+0.0100 (H-2) → L
**VWN**	2.321 (-0.674)	0.55	3.008 (0.0126)	0.54	0.9838 H→L + 0.0141 (H-2) → L
**SAOP**	2.421 (-0.575)	0.54	3.097 (0.102)	0.54	0.9319 H→L + 0.0218 (H-2) → L
**LB94**	2.359 (-0.636)	0.56	2.946 (-0.0487)	0.51	0.9220 H→L + 0.0258 (H-2) → L
**BHandH**	3.503(0.508)	0.62	3.543 (0.548)	0.72	0.9738 H→L + 0.0048 (H-1) → L
**M06-2x**	3.453 (0.458)	0.63	3.373 (0.378)	0.68	0.9700 H→ L + 0.0048 (H-1) → L

Table S10. ΔSCF and TDDFT vertical excitation energies and oscillator strength for the HOMO→LUMO transition of **9**, and for a selection of DFT *xc* potentials. The differences with respect to the experimental value of 2.479 eV is also reported in parenthesis. . The CASPT2/cc-pVDZ value of the vertical excitation energy is 2.479 eV. [1]. For TDDFT the eigenvector corresponding to the lowest energy root is also reported (H: HOMO, L:LUMO).

	**ΔSCF**		**TDDFT**		
**XC**	**ε(eV)**	**f**	**ε(eV)**	**f**	**dominant excitations**
**BLYP**	1.919 (-0.560)	0.52	2.849 (0.370)	0.30	0.5539 H→L + 0.4140 (H-1) →L
**PBE**	1.934 (-0.546)	0.52	2.863 (0.384)	0.30	0.5528 H→L + 0.4169 (H-1) →L
**PW86X**	1.929 (-0.550)	0.25	2.851 (0.372)	0.29	0.5452 H→L + 0.4246 (H-1) →L
**B3LYP**	2.311 (-0.168)	0.59	2.962 (0.483)	0.60	0.9525 H→L + 0.0252 (H-1) →L
**PBE0**	2.415 (-0.0641)	0.61	2.988 (0.509)	0.62	0.9639 H→L + 0.0168 (H-1) →L
**CAM-B3LYP**	-	-	2.963 (0.484)	0.67	0.9765 H→L + 0.0064 (H-1) →L
**wPBEh**	-	-	2.970 0.491)	0.66	0.9766 H→L
**wB97x**	-	-	2.959 (0.480)	0.69	0.9738 H→L
**VWN**	1.917 (-0.562)	0.53	2.842 (0.363)	0.26	0.4813 H→L + 0.4907 (H-1) →L
**SAOP**	2.007 (-0.472)	0.55	2.886 (0.407)	0.42	0.7535 H→L + 0.2107 (H-1) →L
**LB94**	1.946 (-0.533)	0.55	2.745 (0.266)	0.17	0.3531 H→L + 0.6214 (H-1) →L
**BHandH**	2.868 (0.389)	0.67	3.014 (0.535)	0.70	0.9797 H→L + 0.051 (H-1) →L
**M06-2X**	-	-	2.869 (0.390)	0.66	0.9781 H→ L + 0.056 (H-1) →L

Table S11. ΔSCF and TDDFT vertical excitation energies and oscillator strength for the HOMO→LUMO transition of **10**, and for a selection of DFT *xc* potentials. The differences with respect to the experimental value of 2.963 eV is also reported in parenthesis. The CASPT2/cc-pVDZ value of the vertical excitation energy is 3.055 eV [1]. For TDDFT the eigenvector corresponding to the lowest energy root is also reported (H: HOMO, L:LUMO).

	**ΔSCF**		**TDDFT**		
**XC**	**ε(eV)**	**f**	**ε(eV)**	**f**	**dominant excitations**
**BLYP**	2.615 (-0.348)	0.40	3.237 (0.274)	0.28	0.8410 H→L + 0.1025 (H-2) →L
**PBE**	2.643 (-0.320)	0.40	3.259 (0.296)	0.28	0.8408 H→L + 0.1063 (H-2) →L
**PW86X**	2.621 (-0.342)	0.40	3.243 (0.280)	0.28	0.8414 H→L + 0.1016 (H-2) →L
**B3LYP**	2.847 (-0.116)	0.41	3.418 (0.455)	0.39	0.9529 H→L + 0.0282 (H-2) →L
**PBE0**	2.910 (-0.0533)	0.41	3.477 (0.514)	0.40	0.9605 H→L + 0.0216 (H-2)→L
**CAM-B3LYP**	3.255 (0.292)	0.42	3.545 (0.582)	0.44	0.9677 H→L + 0.0055 (H-2) →L
**wPBEh**	-	-	3.576 (0.613)	0.43	0.9668 H→L
**wB97x**	-	-	3.629 (0.666)	0.45	0.9598 H→L
**VWN**	2.702 (-0.261)	0.41	3.236 (0.273)	0.28	0.8461 H→L + 0.1028 (H-2) →L
**SAOP**	2.684 (-0.279)	0.42	3.281 (0.318)	0.31	0.8850 H→L + 0.0877 (H-2) →L
**LB94**	2.653 (-0.310)	0.42	3.129 (0.166)	0.27	0.8514 H→L + 0.1064 (H-2) →L
**BHandH**	3.223 (0.260)	0.42	3.609 (0.646)	0.46	0.9714 H→L + 0.0050 (H-2) →L
**M06-2X**	3.388 (0.425)	0.45	3.462 (0.499)	0.42	0.9685 H→L+ 0.0044 (H-2) →L

Table S12. ΔSCF and TDDFT vertical excitation energies and oscillator strength for the HOMO→LUMO transition of **11**, and for a selection of DFT *xc* potentials. The differences with respect to the experimental value of 2.109 eV is also reported in parenthesis. The CASPT2/cc-pVDZ value of the vertical excitation energy is 1.957 eV [1]. For TDDFT the eigenvector corresponding to the lowest energy root is also reported (H: HOMO, L:LUMO).

	**ΔSCF**		**TDDFT**		
**XC**	**ε(eV)**	**f**	**ε(eV)**	**f**	**excitation**
**BLYP**	1.737 (-0.372)	0.43	2.422 (0.313)	0.12	0.4222 H→L + 0.5674 (H-1) →L
**PBE**	1.751 (-0.359)	0.44	2.424 (0.315)	0.12	0.4118 H→L + 0.5780 (H-1) →L
**PW86X**	1.744 (-0.365)	0.43	2.415 (0.306)	0.12	0.4171 H→L + 0.5726 (H-1) →L
**B3LYP**	2.055 (-0.0537)	0.48	2.625 (0.516)	0.38	0.8224 H→L + 0.1668 (H-1) →L
**PBE0**	2.133 (0.0244)	0.49	2.658 (0.549)	0.43	0.8742 H→L + 0.1143 (H-1) →L
**CAM-B3LYP**	2.548 (0.439)	0.53	2.648 (0.539)	0.55	0.9578 H→L + 0.0227 (H-1) →L
**wPBEh**	2.603 (0.494)	0.53	2.660 (0.551)	0.55	0.9601 H→L + 0.0186 (H-1) →L
**wB97x**	2.903 (0.794)	0.56	2.634 (0.525)	0.59	0.9655 H→L
**VWN**	1.746 (-0.363)	0.44	2.380 (0.271)	0.11	0.4069 H→L + 0.5830 (H-1) →L
**SAOP**	1.799 (-0.310)	0.45	2.478 (0.369)	0.18	0.5426 H→L + 0.4466 (H-1) →L
**LB94**	1.764 (-0.345)	0.45	2.332 (0.223)	0.12	0.4598 H→ L + 0.5293 (H-1) →L
**BHandH**	2.508 (0.399)	0.53	2.684 (0.575)	0.57	0.9634 H→L + 0.0201 (H-1) →L
**M06-2X**	2.629 (0.520)	0.57	2.558 (0.449)	0.54	0.9611 H→L+ 0.0204 (H-1) →L

Table S13. ΔSCF and TDDFT vertical excitation energies and oscillator strength for the HOMO→LUMO transition of **12**, and for a selection of DFT *xc* potentials. The differences with respect to the experimental value of 2.755 eV is also reported in parenthesis. The CASPT2/cc-pVDZ value of the vertical excitation energy is 2.693 eV [1]. For TDDFT the eigenvector corresponding to the lowest energy root is also reported (H: HOMO, L:LUMO).

	**ΔSCF**		**TDDFT**		
**XC**	**ε(eV)**	**f**	**ε(eV)**	**f**	**dominant excitations**
**BLYP**	2.438 (-0.317)	0.31	2.851 (0.0958)	0.13	0.8604 H^-→L + 0.1246 → (L+1)
**PBE**	2.449 (-0.306)	0.31	2.866 (0.111)	0.13	0.8596 H→L + 0.1256 H → (L+1)
**PW86X**	2.426 (-0.329)	0.31	2.852 (0.0965)	0.13	0.8588 H→L + 0.1264 H → (L+1)
**B3LYP**	2.702 (-0.0528)	0.33	3.173 (0.418)	0.25	0.9423 H→L + 0.0482 H → (L+1)
**PBE0**	2.745 (-0.0097)	0.33	3.256 (0.501)	0.27	0.9532 H→L + 0.0360 H → (L+1)
**CAM-B3LYP**	3.087 (0.332)	0.33	3.428 (0.673)	0.37	0.9648 H→L + 0.0049 H → (L+1)
**wPBEh**	-	-	3.451(0.696)	0.37	0.9611 H→L + 0.0143 (H-1) → (L+1)
**wB97x**	3.376(0.621)	0.33	3.548 (0.793)	0.41	0.9493 H→L + 0.0226 (H-1) → (L+1)
**VWN**	2.509 (-0.246)	0.32	2.843 (0.088)	0.13	0.8563 H→L + 0.1289 H → (L+1)
**SAOP**	2.526 (-0.229)	0.33	2.922 (0.167)	0.15	0.8788 H→L + 0.1058 H → (L+1)
**LB94**	2.467 (-0.288)	0.33	2.751 (-0.0042)	0.13	0.8520 H→L + 0.1325 H → (L+1)
**BHandH**	3.086 (0.331)	0.34	3.496 (0.741)	0.38	0.9685 H→L + 0.0045 H → (L+1)
**M06-2X**	3.292 (0.537)	0.38	3.343 (0.588)	0.36	0.9645 H→L + 0.0125 (H-1) → (L+1)

Table S14. ΔSCF and TDDFT vertical excitation energies and oscillator strength for the HOMO→LUMO transition of **13**, and for a selection of DFT *xc* potentials. The differences with respect to the experimental value of 2.412 eV is also reported in parenthesis [1]. For TDDFT the eigenvector corresponding to the lowest energy root is also reported (H: HOMO, L:LUMO).

	**ΔSCF**		**TDDFT**		
**XC**	**ε(eV)**	**f**	**ε(eV)**	**f**	**dominant excitations**
**BLYP**	2.027 (-0.382)	0.47	2.760 (0.348)	0.25	0.6340 H→L + 0.3383 (H-1) →L
**PBE**	2.042 (-0.37)	0.48	2.774 (0.362)	0.26	0.6510 H→L + 0.3223 (H-1) →L
**PW86X**	2.029 (-0.383)	0.47	2.765 (0.353)	0.25	0.6511 H→L + 0.3223 (H-1) →L
**B3LYP**	2.313 (-0.0989)	0.50	2.921 (0.509)	0.49	0.9262 H→L + 0.0580 (H-1) →L
**PBE0**	2.371 (-0.0406)	0.50	2.950 (0.538)	0.52	0.9457 H→L + 0.0380 (H-1) →L
**CAM-B3LYP**	2.784 (0.372)	0.54	2.938 (0.526)	0.58	0.9676 H→L + 0.0081 (H-1) →L
**wPBEh**	2.840 (0.428)	0.54	2.950 (0.538)	0.58	0.9676 H→L
**wB97x**	3.129 (0.717)	0.56	2.932 (0.520)	0.60	0.9649 H→L
**VWN**	2.088 (-0.324)	0.49	2.757 (0.345)	0.26	0.6739 H→L + 0.3003 (H-1) →L
**SAOP**	2.080 (-0.332)	0.48	2.777 (0.365)	0.26	0.6468 H→L + 0.3255 (H-1) →L
**LB94**	2.055 (-0.357)	0.48	2.635 (0.223)	0.18	0.5360 H→L + 0.4283 (H-1) →L
**BHandH**	2.741 (0.329)	0.54	2.985 (0.573)	0.60	0.9709 H→L + 0.0074 (H-1) →L
**M06-2X**	2.896 (0.484)	0.59	2.822 (0.410)	0.56	0.9693 H→L+ 0.0069 (H-2) →L

Table S15. ΔSCF and TDDFT vertical excitation energies and oscillator strength for the HOMO→LUMO transition of **14**, and for a selection of DFT *xc* potentials. The differences with respect to the experimental value of 2.353 eV is also reported in parenthesis. The CASPT2/cc-pVDZ value of the vertical excitation energy is 2.448 eV [1]. For TDDFT the eigenvector corresponding to the lowest energy root is also reported (H: HOMO, L:LUMO).

	**ΔSCF**		**TDDFT**		
**XC**	**ε(eV)**	**f**	**ε(eV)**	**f**	**dominant excitations**
**BLYP**	2.163 (-0.190)	0.41	2.944 (0.591)	0.18	0.5872 H→L + 0.3739 (H-2) →L
**PBE**	2.185 (-0.168)	0.41	2.959 (0.606)	0.17	0.5738 H→L + 0.3863 (H-2) →L
**PW86X**	2.165 (-0.188)	0.41	2.945 (0.592)	0.17	0.5794 H→L + 0.3799 (H-2) →L
**B3LYP**	2.412 (0.0589)	0.44	3.092 (0.739)	0.37	0.8994 H→L + 0.0848 (H-2) →L
**PBE0**	2.476 (0.123)	0.44	3.131 (0.778)	0.39	0.9213 H→L + 0.0626 (H-2) →L
**CAM-B3LYP**	-	-	3.115 (0.762)	0.45	0.9580 H→L + 0.0181 (H-2) →L
**wPBEh**	-	-	3.143 (0.790)	0.44	0.9567 H→L + 0.0182 (H-2) →L
**wB97x**	-	-	3.124 (0.771)	0.47	0.9592 H→L
**VWN**	2.236 (-0.117)	0.41	2.937 (0.584)	0.17	0.5875 H→L + 0.3764 (H-2) →L
**SAOP**	2.220 (-0.133)	0.42	3.005 (0.652)	0.25	0.7567 H→L + 0.2266 (H-2) →L
**LB94**	-	-	2.888 (0.535)	0.22	0.7468 H→L + 0.2338 (H-2) →L
**BHandH**	2.806 (0.453)	0.48	3.167 (0.814)	0.47	0.9363 H→L + 0.0153 (H-2) →L
**M06-2x**	2.977 (0.624)	0.51	3.017 (0.664)	0.43	0.9613 H→L + 0.0153 (H-2) →L

Table S16. ΔSCF and TDDFT vertical excitation energies and oscillator strength for the HOMO→LUMO transition of **15**, and for a selection of DFT *xc* potentials. The differences with respect to the experimental value of 2.422 eV is also reported in parenthesis [1]. For TDDFT the eigenvector corresponding to the lowest energy root is also reported (H: HOMO, L:LUMO).

	**ΔSCF**		**TDDFT**		
**XC**	**ε(eV)**	**f**	**ε(eV)**	**f**	**dominant excitations**
**BLYP**	2.032 (-0.391)	0.45	2.728 (0.306)	0.15	0.5214 H→L + 0.4660 (H-1) →L
**PBE**	2.043 (-0.379)	0.45	2.728 (0.306)	0.14	0.5078 H→L + 0.4798(H-1) →L
**PW86X**	2.030 (-0.392)	0.45	2.721 (0.299)	0.14	0.5148 H→L + 0.4728(H-1) →L
**B3LYP**	2.323 (-0.0994)	0.48	2.929 (0.507)	0.42	0.8533 H→L + 0.1335 (H-1) →L
**PBE0**	2.384 (-0.0381)	0.48	2.965 (0.543)	0.46	0.8895 H→L + 0.0966 (H-1) →L
**CAM-B3LYP**	-	-	2.968 (0.546)	0.58	0.9533 H→L + 0.0231 (H-1) →L
**wPBEh**	-	-	2.984 (0.562)	0.58	0.9530 H→L + 0.0217 (H-1) →L
**wB97x**	-	-	2.971 (0.549)	0.61	0.9579 H→L
**VWN**	2.080 (-0.342)	0.46	2.689 (0.267)	0.13	0.5037 H→L + 0.4842 (H-1) →L
**SAOP**	2.095 (-0.327)	0.47	2.788 (0.366)	0.21	0.6149 H→L + 0.3728 (H-1) →L
**LB94**	2.063 (-0.360)	0.46	2.633 (0.211)	0.15	0.5447 H→L + 0.4439 (H-1) →L
**BHandH**	2.755 (0.333)	0.51	3.019 (0.597)	0.60	0.9603 H→L + 0.0194 (H-1) →L
**M06-2X**	2.907 (0.485)	0.56	2.856 (0.434)	0.56	0.9577 H→L + 0.0195 (H-1) →L

Table S17. ΔSCF and TDDFT vertical excitation energies and oscillator strength for the HOMO→LUMO transition of **16**, and for a selection of DFT *xc* potentials. The differences with respect to the experimental value of 2.317 eV is also reported in parenthesis [1]. For TDDFT the eigenvector corresponding to the lowest energy root is also reported (H: HOMO, L:LUMO).

	**ΔSCF**		**TDDFT**		
**XC**	**ε(eV)**	**f**	**ε(eV)**	**f**	**excitation**
**BLYP**	1.923 (-0.394)	0.46	2.719 (0.402)	0.39	0.8085 H→L + 0.1785 (H-1) →L
**PBE**	1.934 (-0.384)	0.46	2.726 (0.409)	0.39	0.8070 H→L + 0.1802 (H-1) →L
**PW86X**	1.921 (-0.396)	0.46	2.732 (0.415)	0.38	0.8024 H→L + 0.1846 (H-1) →L
**B3LYP**	2.203 (-0.114)	0.49	2.845 (0.528)	0.55	0.9291 H→L + 0.0582 (H-1) →L
**PBE0**	2.259 (-0.0581)	0.49	2.875 (0.558)	0.58	0.9414 H→L + 0.0453 (H-1) →L
**CAM-B3LYP**	2.662 (0.345)	0.52	2.859 (0.542)	0.64	0.9625 H→L + 0.0148 (H-1) →L
**w-PBEh**	2.723 (0.406)	0.51	2.879 (0.562)	0.64	0.9607 H→L + 0.0146 (H-1) →L
**wB97x**	3.007 (0.690)	0.53	2.862 (0.545)	0.66	0.9607 H→L
**VWN**	1.968 (-0.349)	0.48	2.692 (0.375)	0.38	0.8141 H→L + 0.1731 (H-1) →L
**SAOP**	1.990 (-0.327)	0.48	2.757 (0.440)	0.44	0.8497 H→L + 0.1380 (H-1) →L
**LB94**	1.956 (-0.361)	0.47	2.633 (0.316)	0.39	0.8289 H→L + 0.1585 (H-1) →L
**BHandH**	2.611 (0.293)	0.52	2.900 (0.583)	0.67	0.9675 H→L + 0.0128 (H-1) →L
**M06-2X**	2.794 (0.477)	0.57	2.758 (0.441)	0.63	0.9649 H→L + 0.0131 (H-1) →L

Table S18. Weight of the ground-state KS determinant, and of the single-excitations out of the reference KS determinant in the HOMO→LUMO excited SCF wave function, obtained by using the PBE0 *xc* potential. Also reported are the weight of double and higher excitations (h.e.).

	c_0	$c_{HOMO \rightarrow LUMO}$	$\sum_{ia} \lvert c_{ia} \rvert^2$	h.e.
**1**	0.00	0.690	0.478	0.522
**2**	0.00	-0.692	0.482	0.518
**3**	4.47×10^{-2}	-0.677	0.482	0.516
**4**	5.15×10^{-2}	-0.684	0.479	0.518
**5**	-3.50×10^{-2}	0.696	0.486	0.513
**5_h**	-3.42×10^{-2}	0.695	0.488	0.511
**6**	0.00	-0.690	0.477	0.523
**7**	2.86×10^{-2}	0.696	0.485	0.514
**8**	-6.59×10^{-2}	0.695	0.486	0.510
**9**	6.14×10^{-3}	0.696	0.486	0.514
**10**	2.60×10^{-3}	-0.691	0.478	0.522
**11**	0.00	-0.692	0.480	0.520
**12**	0.00	-0.688	0.475	0.525
**13**	0.00	0.692	0.480	0.520
**14**	1.03×10^{-3}	0.691	0.479	0.521
**15**	-1.35×10^{-3}	-0.692	0.481	0.519
**16**	0.00	-0.691	0.479	0.521

Table S19. Weight of the ground-state KS determinant, and of the single-excitations out of the reference KS determinant in the HOMO→LUMO excited SCF wave function, obtained by using the B3LYP *xc* potential. Also reported are the weight of double and higher excitations (h.e.).

	c_0	$c_{HOMO \rightarrow LUMO}$	$\sum_{ia} \lvert c_{ia} \rvert^2$	h.e.
**1**	0.00	-0.694	0.482	0.518
**2**	0.00	0.696	0.486	0.514
**3**	-5.74×10^{-2}	0.673	0.485	0.511
**4**	6.56×10^{-2}	-0.684	0.484	0.512
**5**	3.61×10^{-2}	0.697	0.488	0.511
**5_h**	3.95×10^{-2}	-0.695	0.489	0.509
**6**	0.00	0.693	0.482	0.518
**7**	3.02×10^{-2}	-0.698	0.488	0.511
**8**	5.84×10^{-2}	-0.697	0.488	0.508
**9**	1.64×10^{-2}	0.698	0.488	0.511
**10**	3.67×10^{-3}	0.694	0.482	0.518
**11**	0.00	0.695	0.484	0.516
**12**	0.00	-0.691	0.479	0.521
**13**	0.00	0.695	0.484	0.516
**14**	7.49×10^{-3}	0.694	0.482	0.518
**15**	3.50×10^{-3}	0.695	0.484	0.516
**16**	0.00	0.694	0.483	0.517

References

[1] Momeni, M. R.; Brown, A. Why do TD-DFT Excitation Energies of BODIPY/aza-BODIPY Families Largely Deviate from Experiment? Answers from Electron Correlated and Multireference Methods J. Chem. Theory Comput. **2015**, *11*, 2619-2632.
